# Peroxisome dynamics determines host-derived ROS accumulation and infectious growth of the rice blast fungus

**DOI:** 10.1128/mbio.02381-23

**Published:** 2023-11-15

**Authors:** Jun Zhang, Huimin Li, Wangliu Gu, Kexin Zhang, Xinyu Liu, Muxing Liu, Leiyun Yang, Gang Li, Zhengguang Zhang, Haifeng Zhang

**Affiliations:** 1 Department of Plant Pathology, College of Plant Protection, Nanjing Agricultural University, Key Laboratory of Integrated Management of Crop Diseases and Pests, Ministry of Education, Nanjing, China; 2 The Key Laboratory of Plant Immunity, Nanjing Agricultural University, Nanjing, China; The University of Georgia, Athens, Georgia, USA

**Keywords:** *M. oryzae*-rice interaction, peroxisome dynamics, host-derived ROS, MoKat2, infectious growth

## Abstract

During the early stages of host-pathogen interaction, the production of reactive oxygen species (ROS) in host cells is a critical strategy to halt the spread of pathogens. Pathogens have to minimize the deleterious effects of host-generated ROS for successful invasion. However, the underlying mechanism remains largely elusive. Here, we report that a peroxisomal 3-ketoacyl-CoA thiolase of rice blast fungus *Magnaporthe oryzae*, MoKat2, functions in host ROS homeostasis likely through regulating fungal peroxisome morphology during infection. *M. oryzae* peroxisome elongates in response to ROS stress, and this is attributed to the function of peroxisome localized MoKat2. MoKat2 forms homodimer *in vivo* while the homodimer is dissociated into monomer in response to host-derived ROS. The MoKat2 monomer binds to peroxisome membranes through its amphipathic helices to control peroxisome elongation, resulting in host ROS depletion and subsequent *M. oryzae* invasion in rice cells. Our findings shed light on the interplay between pathogenic peroxisome dynamics and host ROS accumulation, expanding the crucial role of organelle-mediated infection in plant-microbe interactions.

**IMPORTANCE:**

The interplay between plant and pathogen is a dynamic process, with the host’s innate defense mechanisms serving a crucial role in preventing infection. In response to many plant pathogen infections, host cells generate the key regulatory molecule, reactive oxygen species (ROS), to limit the spread of the invading organism. In this study, we reveal the effects of fungal peroxisome dynamics on host ROS homeostasis, during the rice blast fungus *Magnaporthe oryzae* infection. The elongation of the peroxisome appears contingent upon ROS and links to the accumulation of ROS within the host and the infectious growth of the pathogen. Importantly, we identify a peroxisomal 3-ketoacyl-CoA thiolase, MoKat2, responsible for the elongation of the peroxisome during the infection. In response to host-derived ROS, the homodimer of MoKat2 undergoes dissociation to bind peroxisome membranes for peroxisome elongation. This process, in turn, inhibits the accumulation of host ROS, which is necessary for successful infection. Overall, our study is the first to highlight the intricate relationship between fungal organelle dynamics and ROS-mediated host immunity, extending the fundamental knowledge of pathogen-host interaction.

## INTRODUCTION

Plants have evolved multilayered innate immune systems during the long process of host-pathogen interactions. The activated defense responses include protein kinase signaling pathway, reactive oxygen species (ROS) burst, callose accumulation, and the expression of pathogenesis-related genes (1, 2). ROS produced by host cells plays an important role in triggering immunity and limiting pathogen infection in the early stage of pathogen infection (3, 4). Correspondingly, pathogens have evolved ingenious mechanisms to overcome host immunity by eliminating ROS or suppressing host ROS generation through secreting a large number of catalases, peroxidases, superoxide, and effectors (5–8).

Extracellular ROS-degrading enzymes play crucial roles in pathogen survival in host cells. In the rice blast fungus *Magnaporthe oryzae*, the basic leucine zipper transcription factors MoAp1 and MoAtf1 are important for ROS scavenging by directly or indirectly regulating the expression of a series of extracellular catalase-peroxidase encoding genes (9, 10). Protein phosphatase MoYvh1 is translocated into the nucleus under oxidative stress conditions and also plays a role in ROS scavenging by regulating the activity of laccases and peroxidases (11, 12). The COPII cargo receptor MoErv29 regulates the secretion of extracellular redox enzymes to eliminate host-derived ROS (13). The obligate biotrophic fungal pathogen of barley, *Blumeria graminis* f. sp. *hordei*, secretes an extracellular catalase B to scavenge ROS during infection (14). Meanwhile, pathogens are able to secrete a large number of extracellular effectors to suppress host immunity. In *M. oryzae*, Auxilin-like protein MoSwa2 promotes the secretion of many extracellular redox proteins, such as apoplastic effector MoSef1 to inhibit host immunity and ROS generation (15). MoErv29 promotes the secretion of apoplastic effectors MoSlp1 and MoEcp1 to suppress host immunity (13). Apoplastic ascorbate oxidase MoAo1 plays a critical role in ROS-mediated innate host immunity by regulating the apoplast redox state in rice cells (16). Several key avirulence factors AvrPiz-t, AvrPii, and Avr-Pita target a number of rice proteins to suppress host ROS generation and metabolism (17–22). In the corn smut fungus *Ustilago maydis*, effector Pep1 suppresses ROS production by inhibition of host peroxidase activity (23). The lectin fungal glucan binding 1, secreted from the root endophyte *Piriformospora indica*, specifically interacts with β-1, 6-linked glucan, altering cell wall composition and suppressing glucan-triggered ROS production in plants (24). Besides, the antioxidation systems dependent on glutathione and thioredoxin are also important for *M. oryzae* infection to neutralize host-generated ROS (25). Although different regulatory pathways and extracellular proteins of pathogens have been identified to overcome host immunity mediated by host-derived ROS, the underlying mechanism in this critical process remains largely elusive and understudied.

Organelles, such as peroxisome and mitochondrion, are important for various cellular processes in eukaryotes. Some organelles undergo quick dynamics that are critical for infectious growth during penetration (26, 27). Peroxisome is a ubiquitous single membrane-bound organelle in eukaryotes, and it plays an important role in various activities of organisms, including fatty acid β-oxidation, degradation of hydrogen peroxide, and biosynthesis of ether lipids (28). In response to cellular changes, peroxisome is able to alter the morphology, number, and the associated protein composition, and this dynamic is necessary to maintain normal activities of organisms (29, 30). Peroxisomes can also play an important role in cellular redox homeostasis that in turn regulates cell metabolic pathways (31, 32). It has been demonstrated that peroxisomes can rapidly sense changes in their environment and modify their metabolism and dynamics, all of which are regulated by ROS (33–35). In plants and mammals, ROS induced by pathogen infection, trauma, osmosis, and drought, as well as exogenous hydrogen peroxide treatment, leads to the dynamics of tubular extensions from torus-like organelles peroxisomes and increased expression of biosynthesis-related genes, thus maintaining the adaptability of organisms to different stresses (34, 36). Additionally, peroxisomes have been shown to change their morphology dramatically in response to ROS stress, and peroxisomal extensions were named as peroxules or JEP (juxtaposed elongated peroxisome) (37, 38). These findings suggest that peroxisome dynamics plays important regulatory roles in response to intracellular and extracellular ROS to maintain cellular redox homeostasis in plants and mammals. However, whether and how peroxisome dynamics contribute to pathogen infection during pathogen-host interaction have not been reported yet.

In this study, adopting the *M. oryzae*-rice interaction system, we demonstrated that *M. oryzae* peroxisome undergoes dynamic changes during infection, which is host-derived ROS dependent, and its elongation is essential for host ROS accumulation and infectious growth. We further identified the peroxisomal 3-ketoacyl-CoA thiolase MoKat2 as a key regulator involved in this process. In addition, we found this regulatory process is dependent on MoKat2 monomer that is dissociated from its dimer in response to host-derived ROS during early infection stage. The MoKat2 monomer accumulates onto peroxisome membrane through the exposed amphipathic helix (AH) domains responsible for membrane curvature, to control the elongation of peroxisome, thereby suppressing the accumulation of host ROS to facilitate infectious growth.

## RESULTS

### *M. oryzae* peroxisome undergoes elongation during early infection stage to repress host ROS accumulation

To examine whether peroxisomes exhibit dynamics during fungal pathogen infection and colonization, we expressed a peroxisome marker protein Pex14 (39) fused with green fluorescent protein in the wild-type Guy11 (Guy11/Pex14-GFP). Conidial suspensions of Guy11/Pex14-GFP were inoculated into rice sheaths of CO-39 and K14, and the peroxisome morphology in invasive hyphae (IH) was examined under a confocal microscope in a time course experiment. In IH of both CO-39 and K14 cells, a portion of elongated peroxisomes was observed at 24 and 48 hpi, and almost all peroxisomes were punctate in IH at 72 hpi. In IH of CO-39 cells, 50.0% and 47.7% peroxisomes exhibited tubular shape at 24 and 48 hpi, respectively (Fig. S1A and C). Similarly, in IH of K14 cells, 60.6% and 44.2% peroxisomes exhibited tubular shape at 24 and 48 hpi, respectively (Fig. S1B and C). These findings suggested that peroxisomes of *M. oryzae* undergo morphology alteration during infection, and tubular peroxisomes occur during the early infection phases (24–48 hpi) of *M. oryzae*.

**Fig 1:**
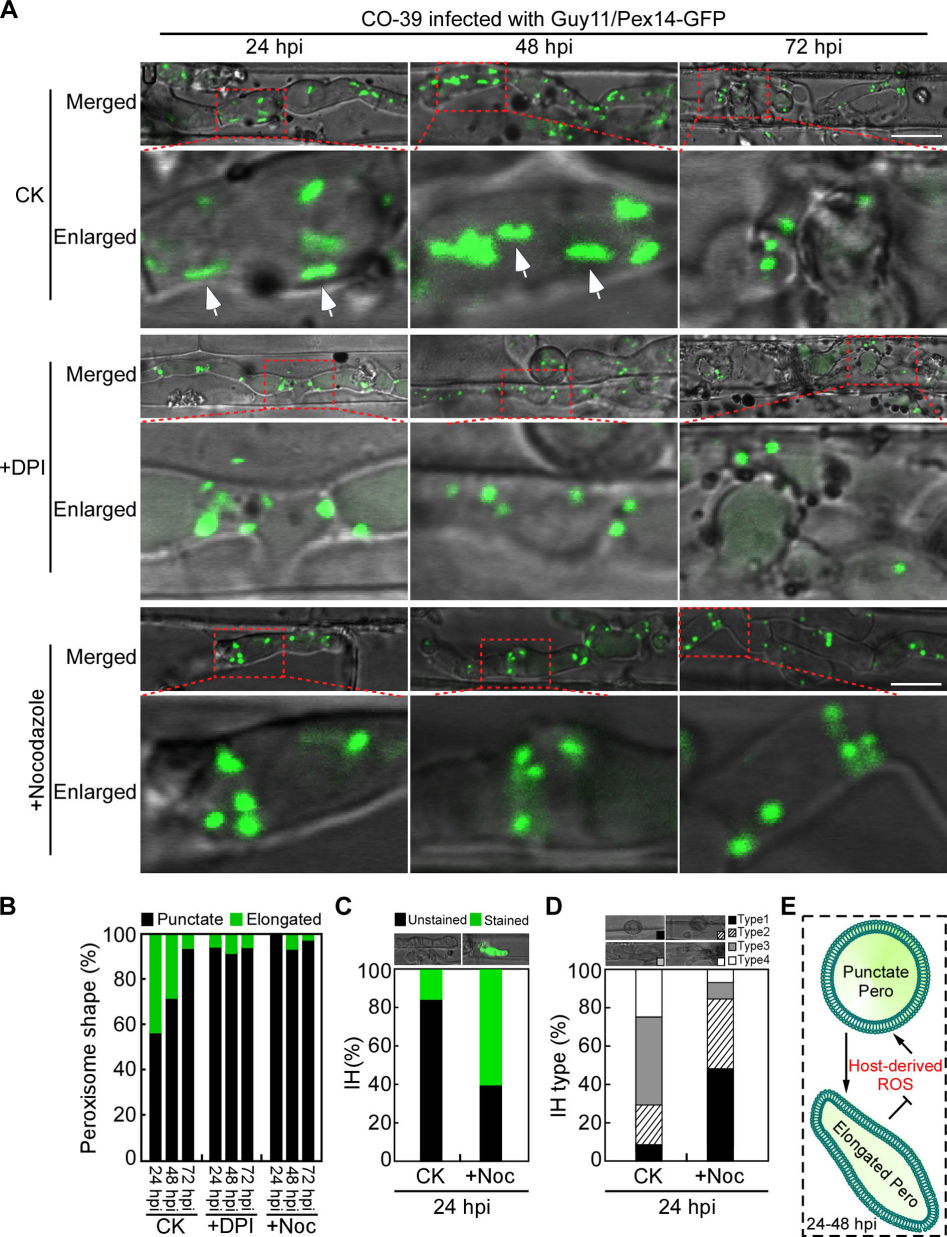
*M. oryzae* peroxisome undergoes elongation during early infection stage to repress host ROS accumulation. (A) Conidial suspensions of Guy11/Pex14-GFP strain were injected into detached rice sheaths of cultivar CO-39 with or without DPI or nocodazole (Noc) treatment; and peroxisome morphology in IH was examined at 24, 48, and 72 hpi, respectively. Bar = 10 µm. White arrows indicate the elongated peroxisomes. (B) Statistical analysis of the percentage of peroxisome shape in IH. (C) Conidial suspensions of Guy11/Pex14-GFP strain pre-treated with nocodazole were injected into detached rice sheaths of cultivar CO-39 and stained by CM-H_2_DCFDA at 24 hpi. Percentage of IH stained or unstained by CM-H_2_DCFDA was statistically analyzed. (D) Conidial suspensions of Guy11/Pex14-GFP strain treated with or without nocodazole were injected into detached rice sheaths and statistically analyzed the IH type at 24 hpi (type 1: no infectious hyphae; type 2: one infectious hyphae; type 3: two or three branches restricted in one cell; and type 4: more than three branches extended to a neighboring cell). (E) Scheme highlighting the regulatory relationship between peroxisome (Pero) morphology and host-derived ROS.

Upon *M. oryzae* infection, rice cells generate a large amount of ROS to restrict invasive hyphae growth at the early infection stage (24–48 hpi) (8), which is correlated with the occurrence of tubular peroxisome in *M. oryzae*. Therefore, we hypothesized that peroxisome dynamics during infection is host-derived ROS dependent. To test this possibility, we examined peroxisome morphology of IH in rice cells treated with or without diphenyleneiodonium (DPI), the NADPH oxidase inhibitor. The results showed that the percentage of tubular peroxisomes was obviously decreased when treated with DPI compared to the untreated control. It was 44.3% and 29.1% in control at 24 and 48 hpi, and decreased to 7.0% and 9.3% under DPI treatment, which was similar to that observed at 72 hpi (Fig. 1A), indicating that peroxisome dynamics during infection is dependent on host-derived ROS. To investigate whether the elongation of peroxisome is essential for ROS scavenging, we treated *M. oryzae* with nocodazole, a chemical that blocks the self-assembly of tubulin, leading to microtubule depolymerization which is required for JEP formation (38), and examined the peroxisome morphology and ROS accumulation in rice cells by CM-H_2_DCFDA staining, an indicator for cellular ROS (40). The percentage of tubular peroxisomes decreased to 0 and 8.2% at 24 and 48 hpi (Fig. 1A and B). ROS examination revealed that 18% cells exhibited green fluorescence in the non-treated strain infected samples at 24 hpi while this number increased to 60% after nocodazole treatment (Fig. 1C). We further observed the infectious growth of Guy11/Pex14-GFP with or without nocodazole treatment, and found that 48.3% IH was type 1 and 36.7% type 2 under nocodazole treatment, while 46% IH was type 3 and 24% type 4 in the untreated control (Fig. 1D). These results suggested that peroxisome dynamics in IH is host-derived ROS dependent, and its elongation is essential for ROS depletion and infectious growth in *M. oryzae* (Fig. 1E).

### *MoKAT2* encodes a peroxisome 3-ketoacyl-CoA thiolase and it is transcriptionally induced responding to H_2_O_2_

**Fig 2:**
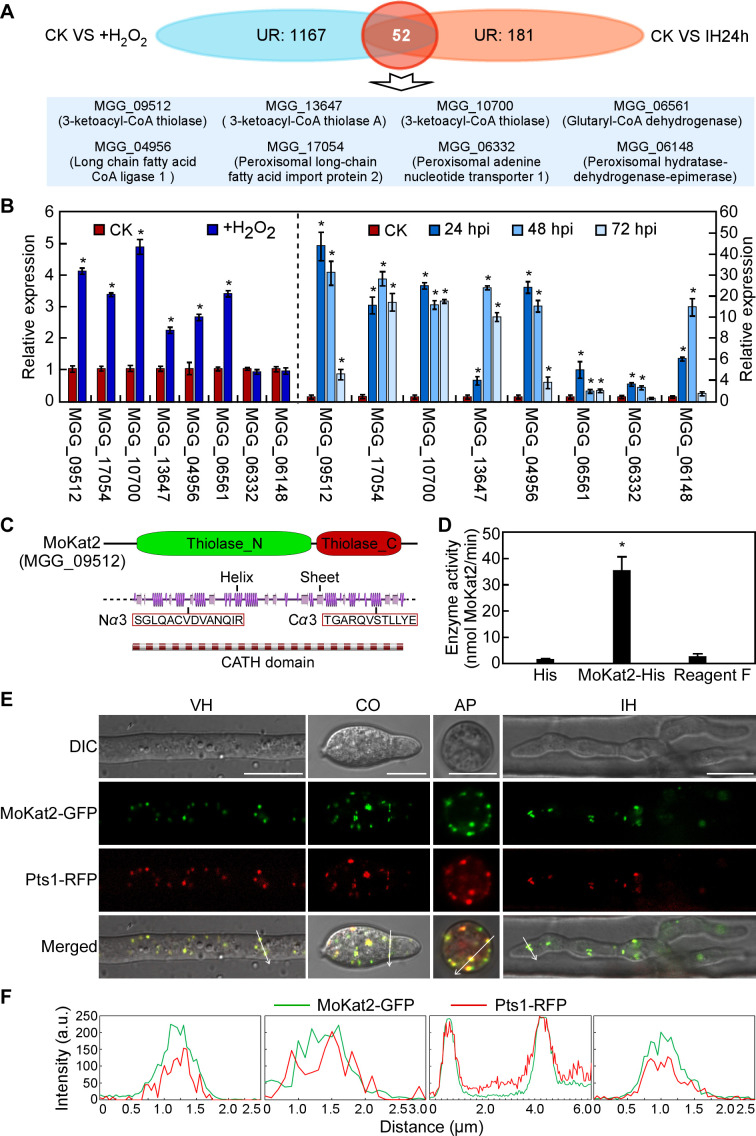
*MoKAT2* encodes a peroxisome 3-ketoacyl-CoA thiolase and it is transcriptionally induced responding to H_2_O_2_. (A) Venn diagram showing the gene numbers of upregulated under H_2_O_2_ treatment (CK VS + H_2_O_2_), or in early invasive hyphae (CK VS IH24h). (B) qRT-PCR analysis of the expression level of *MoKAT2* in vegetative hyphae with or without H_2_O_2_ treatment or during different infectious stages of *M. oryzae*. Error bars are standard deviations from three biological repeats, and asterisk indicates significant differences at *P* < 0.01. CK, vegetative hyphae without treatment. (C) Prediction of the secondary structure of MoKat2 by JPred 4 (http://www.compbio.dundee.ac.uk/jpred4/index.html). N*α*3 and C*α*3: N-terminal and C-terminal amphipathic helices. (D) Ketoacyl-CoA thiolase Activity Assay Kit, colorimetric method assay analysis of the enzyme activity of MoKat2. His and reagent F were used as negative control. Error bars are standard deviations from three biological repeats, and asterisk indicates significant differences at *P* < 0.01. (E) Vegetative hypha (VH), conidium (CO), appressorium (AP), and IH co-expressing MoKat2-GFP and Pts1-RFP were examined under a fluorescence microscope. Bar = 10 µm. (F) Linescan graph analysis of the co-localization of MoKat2-GFP and Pts1-RFP in VH, CO, AP, and IH indicated by white arrows.

To investigate the underlying mechanisms of peroxisome dynamics in response to host-derived ROS, we performed the transcriptome analysis of Guy11 with 5 mM H_2_O_2_ treatment or Guy11 in infecting leaves at 24 hpi (and Guy11 without any treatment as the control) by RNA-Seq experiment (Table S1; Fig. S2A through D). The transcriptomic data revealed a total of 2,543 differentially expressed genes (DEGs; fold change > 2 and *P* value < 0.001), including 1,219 upregulated and 1,324 downregulated genes after 5 mM H_2_O_2_ treatment compared to control check: without H_2_O_2_ treatment (CK); a total of 849 DEGs, including 233 upregulated and 616 downregulated genes in IH at 24 hpi, and 52 genes were upregulated under both H_2_O_2_ treatment and in IH at 24 hpi (Fig. 2A). Kyoto Encyclopedia of Genes and Genomes (KEGG) analysis showed that genes involved in metabolic processes were highly enriched (Fig. S2E through H). Among these 52 genes, we selected six genes that might be relevant to peroxisome functions, including MGG_09512, MGG_13647, MGG_10700, MGG_06561, MGG_04956, and MGG_17054, as well as MGG_06332 and MGG_06148 which only upregulated in IH at 24 hpi (Fig. 2A). We then analyzed the expression level of these eight genes under H_2_O_2_ treatment and on rice leaves at 24 hpi by quantitative RT-PCR (qRT-PCR), and the yielded results were consistent to the RNA-Seq data (Fig. 2B). We further predicted the subcellular localization of these eight genes (https://wolfpsort.hgc.jp/) and found that only MGG_09512 showed peroxisome localization pattern (Table S2-1), and thus we focused on MGG_09512 for further analysis.

MGG_09512 (named MoKat2 hereafter) is the homolog of *Saccharomyces cerevisiae* ScPot1 and *Arabidopsis thaliana* AtKat2, which catalyze a key step in fatty acid β*-*oxidation (41, 42). Structural analysis revealed that MoKat2 contains a thiolase domain and amphipathic helices N*α*3 and C*α*3 in both N-terminal and C-terminal, respectively (Fig. 2C). *In vitro* enzymatic assay showed high thiolase activity of MoKat2 compared to the control (Fig. 2D). We further examined the subcellular localization of MoKat2 in different developmental stages of *M. oryzae*. MoKat2-GFP was co-localized with Pts1-RFP (peroxisomal targeting signal 1) in vegetative hyphae, conidia, appressoria, and IH (Fig. 2E and F). These results suggested that MoKat2 functions as a 3-ketoacyl-CoA thiolase in peroxisome.

### Deletion of *MoKAT2* leads to defects of infectious growth and host ROS accumulation

**Fig 3:**
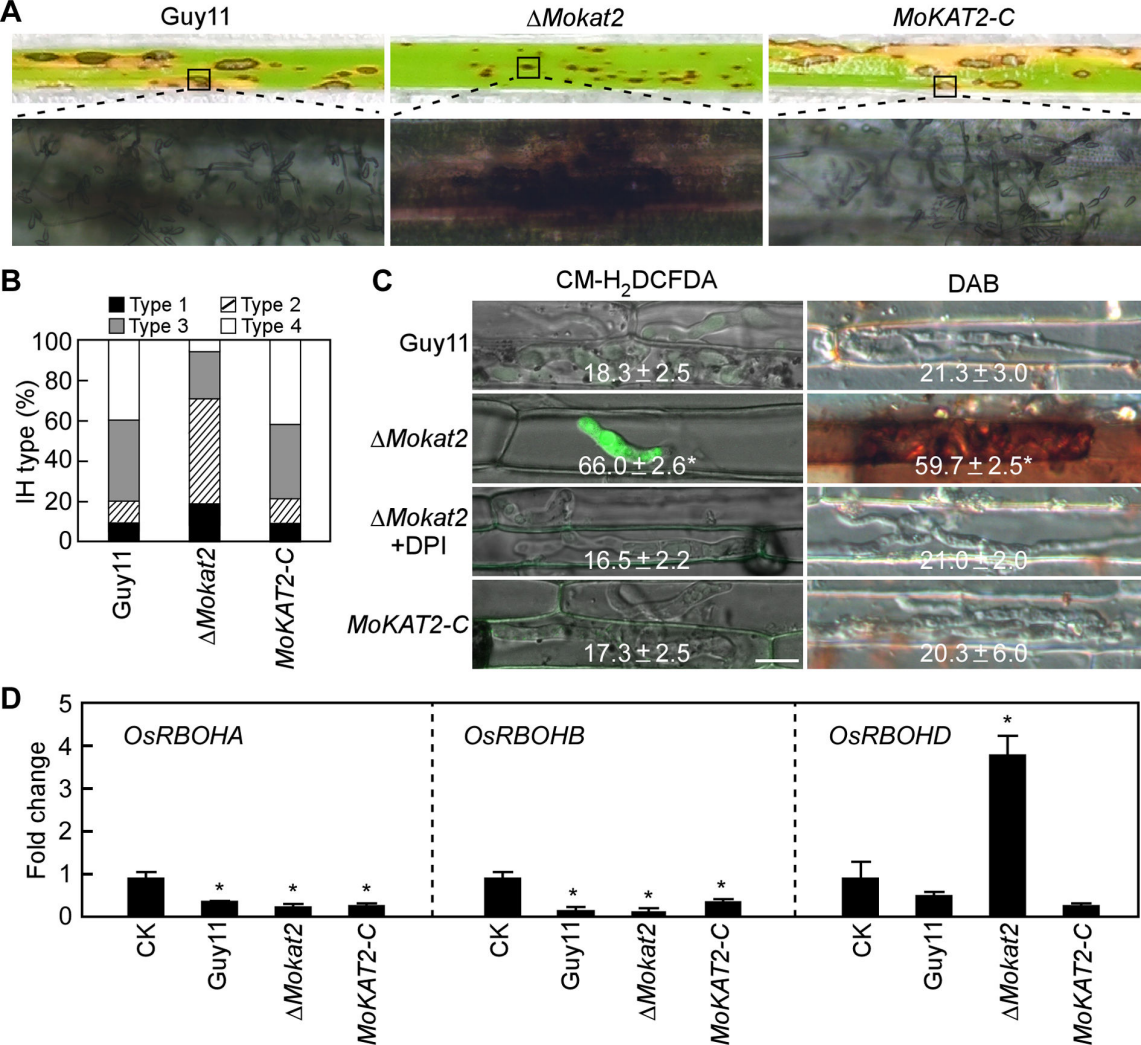
Deletion of *MoKAT2* leads to defects of infectious growth and host ROS accumulation. (A) Spraying assay: conidial suspensions of Guy11, Δ*Mokat2*, and *MoKAT2-C* were sprayed onto susceptible rice seedlings, and photographed at 7 days after incubation (dai) (top panels). Fungal growth assay: the diseased rice leaves were surface-sterilized and incubated in a chamber for 24 h, and examined under a microscope (bottom panels). (B) Conidial suspensions of the indicated strains were injected into detached rice sheaths and statistically analyzed the IH type (refer to Fig. 1D) at 36 hpi. Bar = 10 µm. (C) Rice sheath cells infected by the indicated strains were stained by 5-(and 6-)chloromethyl -2',7'-dichlorodihydrofluorescein diacetate, acetyl ester, used as an indicator for ROS in cells (CM-H_2_DCFDA), or 3,3′-diaminobenzidine (DAB) at 36 hpi with or without DPI treatment, and examined under a confocal microscope; and statistically analyzed the percentage of CM-H_2_DCFDA or DAB staining cells. ±SD (standard deviation) was calculated from three independent experiments, and asterisk indicates significant differences at *P* < 0.01. (D) qRT-PCR analysis of the expression of *OsRBOHA*, *OsRBOHB*, and *OsRBOHD* in rice inoculated with conidial suspensions of Guy11, Δ*Mokat2,* and *MoKAT2*-C at 24 hpi. Error bars are standard deviations from three biological repeats, and asterisk indicates significant differences at *P* < 0.01.

To investigate the biological role of MoKat2 in *M. oryzae*, we generated the ∆*Mokat2* mutant (Fig. S3A and B) and obtained the complemented transformant *MoKAT2*-C by introducing the *MoKAT2*-GFP vector into the mutant. Phenotypic analysis revealed that the growth rate of the ∆*Mokat2* mutant was significantly reduced on minimal medium (MM) media compared with the wild-type Guy11 and *MoKAT2-C* (Table S2-2). Conidial quantification analysis showed that the conidial production was decreased by 24.6% in the mutant. Appressorium formation was not changed, but appressorium turgor was obviously decreased in the mutant due to the defects in translocation and breakdown of lipid droplets (LD) and glycogen during appressorium development (Table S2-2; Fig. S4A and B). Pathogenicity assay revealed that Guy11 and *MoKAT2-C* caused typical lesions that were able to produce numerous conidia on rice leaves, while ∆*Mokat2* caused smaller lesions that failed to produce conidia under the same conditions (Fig. 3A). We further examined the penetration and infectious growth of the mutant on rice sheaths, and found that the IH type of ∆*Mokat2* was 17% type 1, 54.6% type 2, 23% type 3, and 5.3% type 4, in comparison to 9.6% type 1, 11.7% type 2, 40% type 3, and 38.7% type 4 of the wild-type Guy11, with the *MoKAT2-C* strain showing the similar growth pattern to Guy11 (Fig. 3B), indicating that MoKat2 is critical for infectious growth.

We next evaluated the accumulation of host-derived ROS by staining infected rice cells with CM-H_2_DCFDA and found that 66.0% cells infected with the ∆*Mokat2* mutant exhibited green fluorescence, compared to 18.3% and 17.3% of those infected with Guy11 and *MoKAT2-C*, respectively. When treated with DPI, the percentage of cells infected with ∆*Mokat2* that exhibited green fluorescence was decreased to the wild-type level. We also stained the rice cells with DAB (3,3′-diaminobenzidine: a dye used to detect the presence and distribution of ROS in plant cells) and found that 59.7% cells infected with ∆*Mokat2* yielded dark brown precipitate, compared to 21.3% and 20.3% of those infected with Guy11 and *MoKAT2-C*, respectively (Fig. 3C). We then analyzed the superoxide dismutase (SOD) and catalase (CAT) activity which is responsible for ROS scavenging (43), and no obvious difference was found among Guy11, Δ*Mokat2*, and *MoKAT2-C* strains (Fig. S5A and B). We further detected the expression level of the *OsRBOH* genes which are important for ROS generation in rice cells (44, 45) and found that *OsRBOHD* but not *OsRBOHA* and *OsRBOHB* was significantly upregulated in the mutant (Fig. 3D). These results suggested that MoKat2 is essential for infectious growth by suppressing host ROS generation.

### MoKat2 is responsible for peroxisome elongation likely relying on host-derived ROS

**Fig 4:**
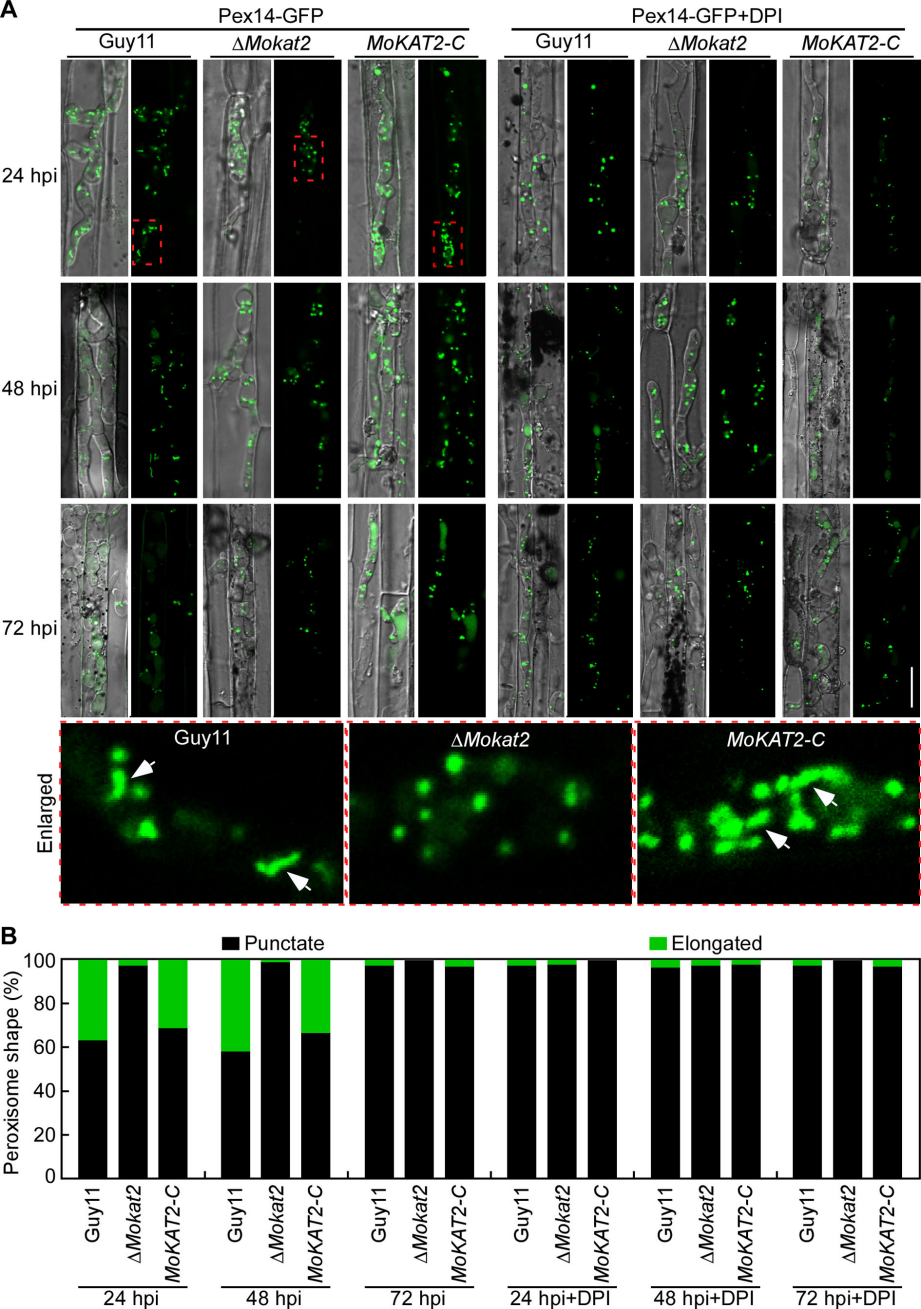
MoKat2 is responsible for peroxisome elongation likely relying on host-derived ROS. (A) Conidial suspensions of Guy11, Δ*Mokat2*, and *MoKAT2-C* expressing Pex14-GFP were injected into detached rice sheaths with or without DPI treatment; and peroxisome morphology in IH was examined at 24, 48, and 72 hpi, respectively. Bar = 10 µm. Enlarged images were viewed by rotating 90° counterclockwise. White arrows indicate the elongated peroxisomes. (B) Statistical analysis of the percentage of peroxisome shape in IH.

To investigate whether MoKat2 plays a role in the regulation of peroxisome dynamics in response to host-derived ROS during infection, we examined the peroxisome morphology in IH of Guy11, ∆*Mokat2*, and *MoKAT2-C* expressing Pex14-GFP at 24, 48, and 72 hpi with or without DPI treatment, respectively. The results revealed that about 36.2% and 31.3% peroxisomes in IH exhibited tubular shape at 24 hpi in Guy11 and *MoKAT2-C*, and 41.9% and 32.9% at 48 hpi, besides 1.37% and 1% with tubular shape at 72 hpi without DPI treatment. Compared to Guy11 and *MoKAT2-C*, only 1.9% and 1.4% peroxisomes exhibited tubular shape at 24 and 48 hpi in the ∆*Mokat2* mutant. Meanwhile, the percentage of tubular peroxisomes was less than 3.2% in IH of Guy11, ∆*Mokat2*, and *MoKAT2-C* at 24, 48, and 72 hpi under DPI treatment (Fig. 4A and B). We further examined the peroxisome morphology in conidia of Guy11, ∆*Mokat2*, and *MoKAT2-C* expressing Pts1-RFP under oxidative stress (H_2_O_2_ and DIA) treatment. The results showed that 70.6% and 76.8% peroxisomes of Guy11 and *MoKAT2-C* exhibited tubular shape under H_2_O_2_ and DIA treatment, and only 9.2% tubular peroxisomes was observed under the same conditions in the ∆*Mokat2* mutant. The percentage of tubular peroxisomes was decreased to 19% and 20% in Guy11 and *MoKAT2-C* simultaneously treated with H_2_O_2_ and antioxidant CAG (catalase of *Aspergillus niger*, a scavenger of H_2_O_2_), which was similar to the ∆*Mokat2* mutant (Fig. S6A and B). These results suggested that MoKat2 plays a critical role in peroxisome elongation in a host-derived ROS-dependent manner during early infection.

### Deletion of *MoKAT2* results in abnormal apoplastic effector secretion

**Fig 5:**
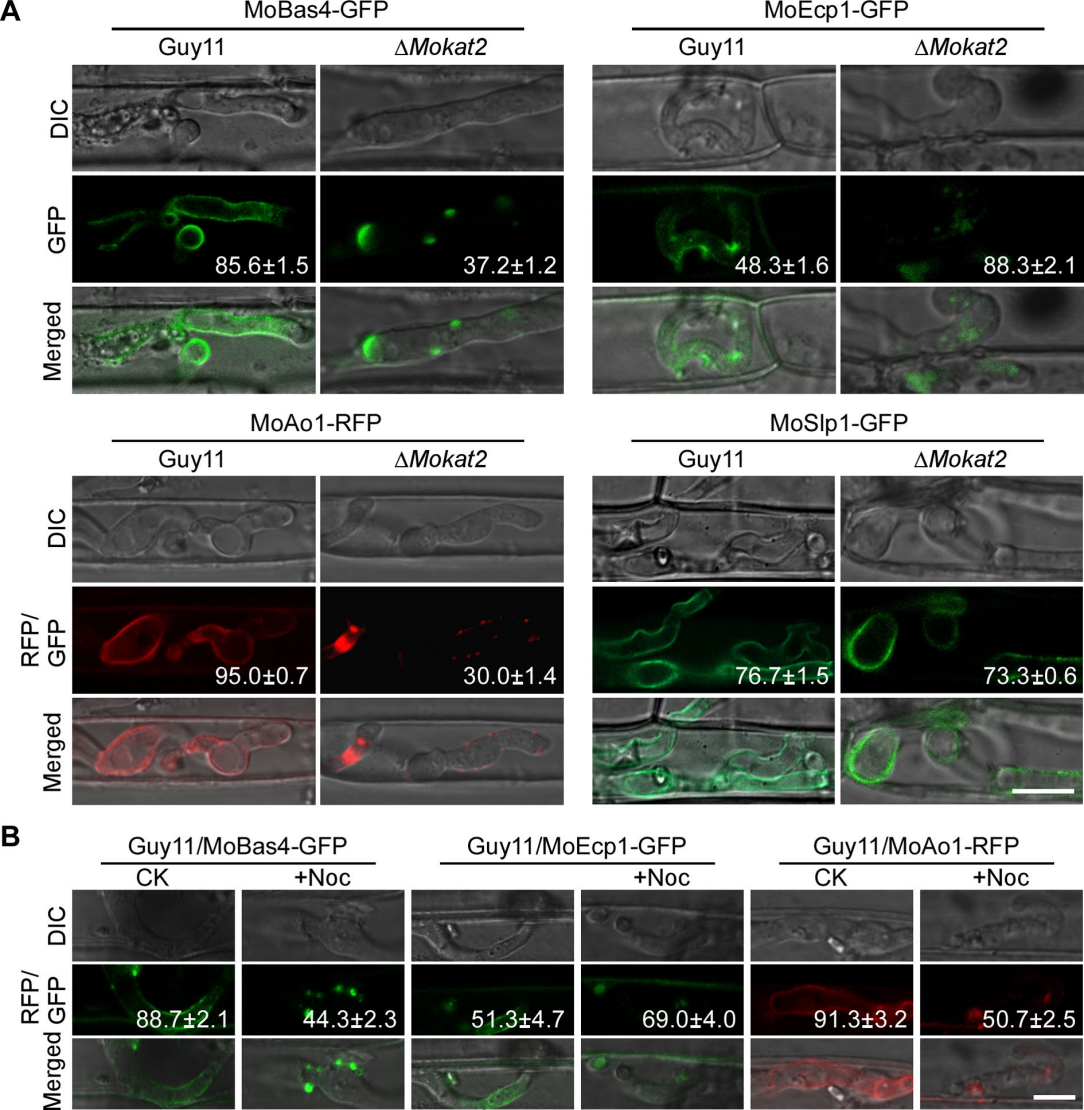
Deletion of *MoKAT2* results in abnormal apoplastic effector secretion. (A) Conidial suspensions of Guy11 and Δ*Mokat2* separately expressing MoBas4-GFP, MoEcp1-GFP, MoAo1-RFP, and MoSlp1-GFP were injected into detached rice sheaths of cultivar CO-39; and the secretion of the indicated proteins was examined at 24 hpi under a fluorescence microscope. The number indicated the percentage of the localization pattern of the corresponding image. Bar = 10 µm. (B) Conidial suspensions of Guy11 separately expressing MoBas4-GFP, MoEcp1-GFP, and MoAo1-RFP pre-treated with nocodazole were injected into detached rice sheaths of cultivar CO-39; and the secretion of the indicated proteins was examined at 24 hpi under a fluorescence microscope. The number indicated the percentage of the localization pattern of the corresponding image. Bar = 10 µm.

To investigate how MoKat2 inhibits host ROS generation, we examined the secretion of several apoplastic and cytoplastic effectors during early infection stage and found that three apoplastic effectors (MoBas4, MoEcp1, and MoAo1) exhibited abnormal secretion pattern in the ∆*Mokat2* mutant compared to the wild-type Guy11. In rice cells infected by ∆*Mokat2*, 37.2% of MoBas4, 88.3% of MoEcp1, and 30.0% of MoAo1 showed abnormal secretion pattern. By contrast, 85.6% of MoBas4, 48.3% of MoEcp1, and 95.0% of MoAo1 showed typical extra-invasive hyphal membrane localization pattern in the cells infected by Guy11 (Fig. 5A). However, the secretion of another apoplastic effector MoSlp1 and two cytoplastic effectors (MoAVR-Pia and MoPwl2) showed no obvious difference in both ∆*Mokat2* and Guy11 (Fig. 5A; Fig. S5C). These results suggested that MoKat2 properly regulates the secretion of apoplastic effectors to suppress host ROS generation during early infection. We further examined the secretion of MoBas4, MoEcp1, and MoAo1 in Guy11 under nocodazole treatment. The results revealed that 44.3% of MoBas4, 69.0% of MoEcp1, and 50.7% of MoAo1 in Guy11 treated with nocodazole exhibited abnormal secretion pattern (Fig. 5B), indicating that peroxisome elongation of *M. oryzae* involves in the secretion of apoplastic effectors. Taken together, we conclude that MoKat2 mediates peroxisome elongation and regulates the secretion of apoplastic effectors, thereby suppressing the host ROS accumulation.

### Host-derived ROS dissociate MoKat2 dimer during *M. oryzae* early infection

**Fig 6:**
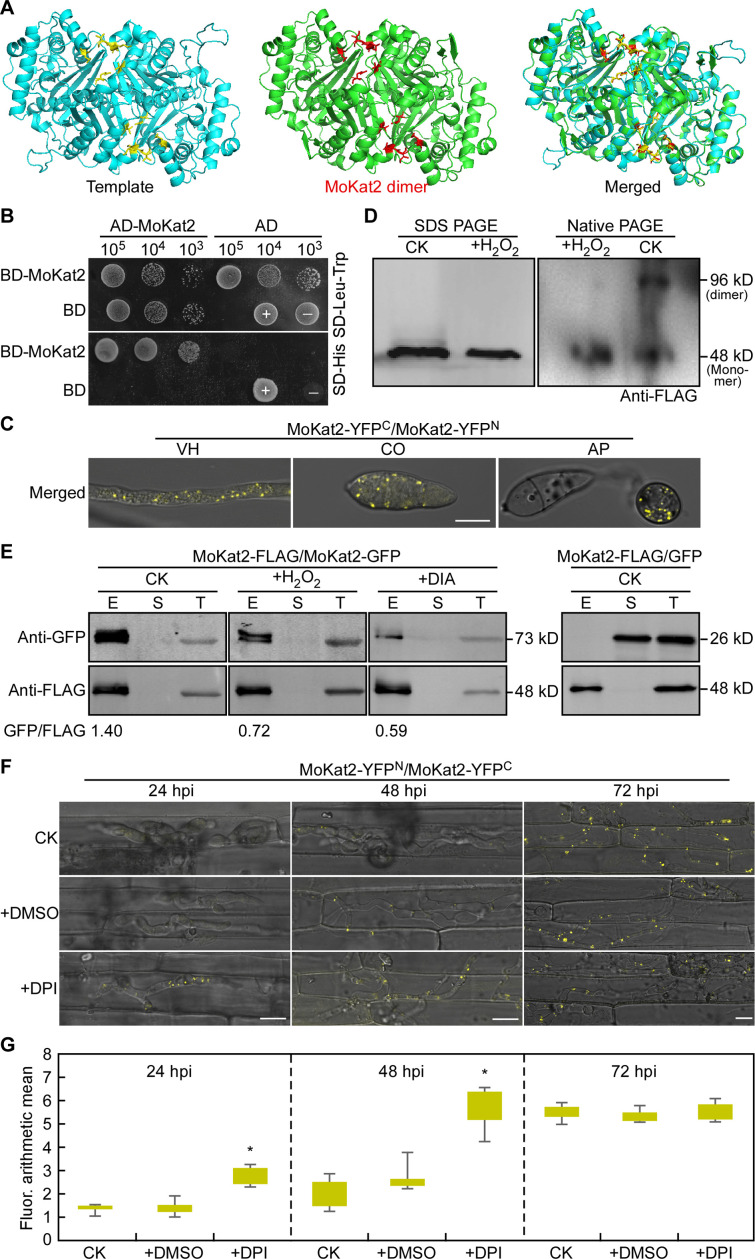
Host-derived ROS dissociate MoKat2 dimer during *M. oryzae* early infection. (A) Homology modeling of MoKat2 in https://swissmodel.expasy.org/. (B) Yeast two-hybrid assay for the interaction of MoKat2 with itself. +: positive control and −: negative control. (C) BIFC analysis of the interaction of MoKat2 with itself. VH: vegetative hypha; CO: conidium; and AP: appressorium. Bar = 10 µm. (D) SDS-PAGE and NativePAGE Bis-Tris Gel System to detect the dissociation of MoKat2 protein under H_2_O_2_ treatment using anti-FLAG antibody. (E) Co-immunoprecipitation assays to detect the interaction intensity of MoKat2 with itself under H_2_O_2_ and DIA treatment using anti-green fluorescent protein (anti-GFP) and anti-FLAG antibodies. (F) Conidial suspensions of Δ*Mokat2* co-expressing MoKat2-YFP^N^ and MoKat2-YFP^C^ were injected into detached rice sheaths with or without DPI treatment; and YFP signals in IH were examined at 24, 48, and 72 hpi, respectively. DMSO, dimethyl sulfoxide; DPI, solvent. Bar = 10 µm. (G) Statistical analysis of the fluorescence arithmetic mean in IH. The fluorescence arithmetic mean and statistical analysis were performed using GraphPad Prism 8.0.1 and ZEN blue, and asterisks represent significant differences at *P* < 0.01.

In *S. cerevisiae* and *A. thaliana*, the dimeric Kat2 catalyzes the conversion of 3-ketoacyl-CoA into acyl-CoA (46, 47). Therefore, we first conducted homology modeling of MoKat2 by SWISS-MODEL (https://swissmodel.expasy.org/) and found that MoKat2 is a homodimer with high global model quality estimate score (50.78%) and high sequence identity (50.78%) to ScPot1. Meanwhile, the binding sites for self-interaction are also well conserved in MoKat2 (Fig. 6A). We then validated the self-interaction of MoKat2 under non-oxidative conditions by Y2H, BiFC (bimolecular fluorescence complementation), NativePAGE western blot, and co-immunoprecipitation (co-IP) assays and proved that MoKat2 formed dimer *in vivo* and *in vitro* (Fig. 6B through E; Fig. S7A). We also examined the self-interaction of MoKat2 under oxidative stress treatment. Both MoKat2 dimer and monomer were detected on NativePAGE gel without H_2_O_2_ treatment while only MoKat2 monomer was detected on NativePAGE gel after H_2_O_2_ treatment or on SDS-PAGE gel in both control and H_2_O_2_ treatment (Fig. 6D). We then analyzed the self-interaction intensity of MoKat2 *in vivo* under oxidative treatment and found that abundance of the eluted MoKat2 protein was obviously decreased under H_2_O_2_ and DIA treatment compared to the untreated control (Fig. 6E), indicating that MoKat2 is dissociated under oxidative conditions.

To further verify whether MoKat2 was dissociated during infection, conidia collected from the transformant co-expressing MoKat2-YFP^N^ and MoKat2-YFP^C^ were inoculated onto the excised leaf sheath, and yellow fluorescence was examined at 24, 48, and 72 hpi. Only very weak yellow fluorescence was observed in IH at 24 and 48 hpi, while strong yellow fluorescence was observed in IH at 72 hpi in control and DMSO treatment. When treated with DPI, strong yellow fluorescence was observed in IH throughout the experiment (Fig. 6F and G; Fig. S7B). We also analyzed the fluorescence intensity in conidia with or without H_2_O_2_ treatment and found that fluorescence intensity was much lower when treated with H_2_O_2_ than the untreated control; when added CAG, fluorescence intensity was recovered to the control level (Fig. S7A, C, and D). These results indicated that MoKat2 is dissociated at early infection stage in response to host-derived ROS.

### The constitutive MoKat2 monomer promotes peroxisome elongation and represses host ROS accumulation

**Fig 7:**
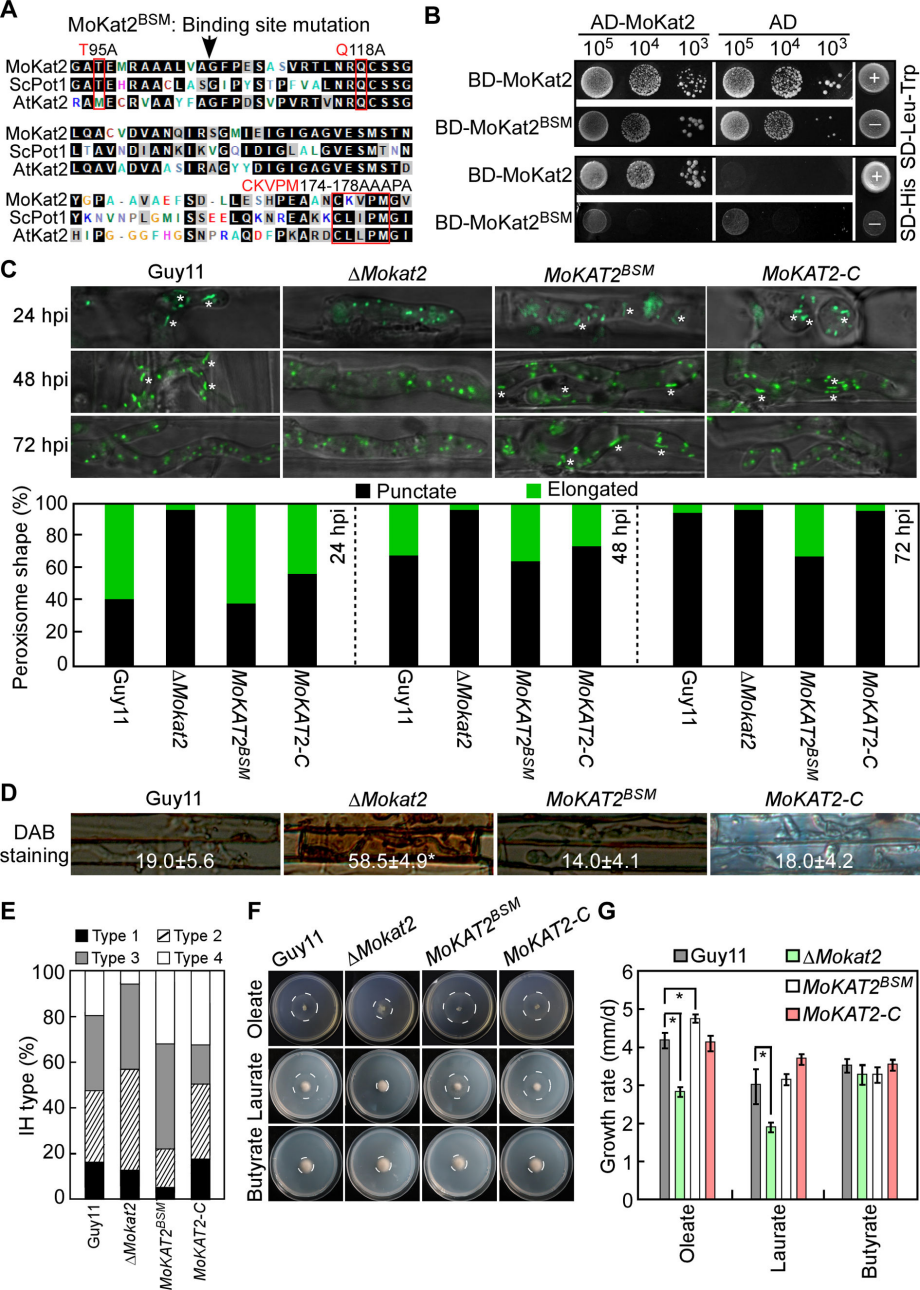
The constitutive MoKat2 monomer promotes peroxisome elongation and represses host ROS accumulation. (A) Conserved binding sites (in red box) were identified in Kat2 homologs from *M. oryzae*, *S. cerevisiae*, and *A. thaliana*. (B) Yeast two-hybrid assay analyses the interaction between MoKat2 and MoKat2^BSM^. (C) Conidial suspensions of the indicated strains were injected into detached rice sheaths. Peroxisome morphology in IH was examined and statistically analyzed at 24, 48, and 72 hpi. White asterisks indicate the elongated peroxisomes. (D) Rice sheath cells infected by the indicated strains were stained by DAB at 24 hpi, and examined under a confocal microscope. The number represents the percentage of DAB staining cells. ±SD was calculated from three independent experiments, and asterisk indicates significant differences at *P* < 0.01. (E) IH type (refer to Fig. 1D) in rice sheaths was statistically analyzed at 24 hpi. (F) The indicated strains were inoculated onto MM medium with oleate, laurate, and butyrate as sole carbon source, respectively, and photographed at 7 dpi. (G) The growth rate was calculated by diameter/days. Error bars are standard deviations from three biological repeats, and asterisk indicates significant differences at *P* < 0.01.

We generated the constitutive MoKat2 monomer construct MoKat2^BSM^ by mutating the conserved binding sites of Kat2 (T95, Q118, CKVPM174-178) (Fig. 7A) and obtained the constitutive MoKat2 monomer transformant *MoKAT2^BSM^*. Yeast two-hybrid assay confirmed that MoKat2^BSM^ was unable to interact with MoKat2 (Fig. 7B). We then examined how *MoKAT2^BSM^* affected peroxisome elongation and ROS accumulation in host cells and found that 62.3% peroxisome in IH of MoKat2^BSM^ exhibited tubular shape and 32.7% with punctate shape at 24 hpi, and 33.6% exhibited tubular shape at 48 hpi. As expected, MoKat2^BSM^ exhibited 31.2% tubular shape at 72 hpi which was much higher than that in Guy11 and *MoKAT2-C* (Fig. 7C). DAB staining revealed that only 14% rice cells infected by MoKat2^BSM^ were stained, very close to Guy11 and *MoKAT2-C* (Fig. 7D). Infectious growth observation revealed that the extension of IH was more severe in MoKat2^BSM^ than that in Guy11 and *MoKAT2-C* (Fig. 7E). These results indicated that constitutive MoKat2 monomer contributes to peroxisome elongation, host ROS accumulation, and infectious growth of *M. oryzae*.

Since MoKat2 is a 3-ketoacyl-CoA thiolase that catalyses a key step in fatty acid β*-*oxidation, we measured the growth rate of Guy11, Δ*Mokat2*, *MoKAT2^BSM^*, and *MoKAT2-C* on plates with short-chain (butyrate <C6), medium-chain (laurate C6-C12), or long-chain (oleate >C12) fatty acids as sole carbon sources. The results showed that the growth rate of Δ*Mokat2* was significantly reduced on oleate and laurate plates compared to Guy11 and *MoKAT2-C*, while the growth rate of *MoKAT2^BSM^* was significantly increased on oleate plates compared to Guy11 and *MoKAT2-C*. No obvious change of these strains was found on other plates (Fig. 7F and G). These results indicated that MoKat2 plays a role in medium- and long-fatty acid oxidation. Constitutive MoKat2 monomer has a higher ability to oxidize long-fatty acids in *M. oryzae*.

### The AH structures of MoKat2 are required for peroxisome elongation in response to oxidative stress

**Fig 8:**
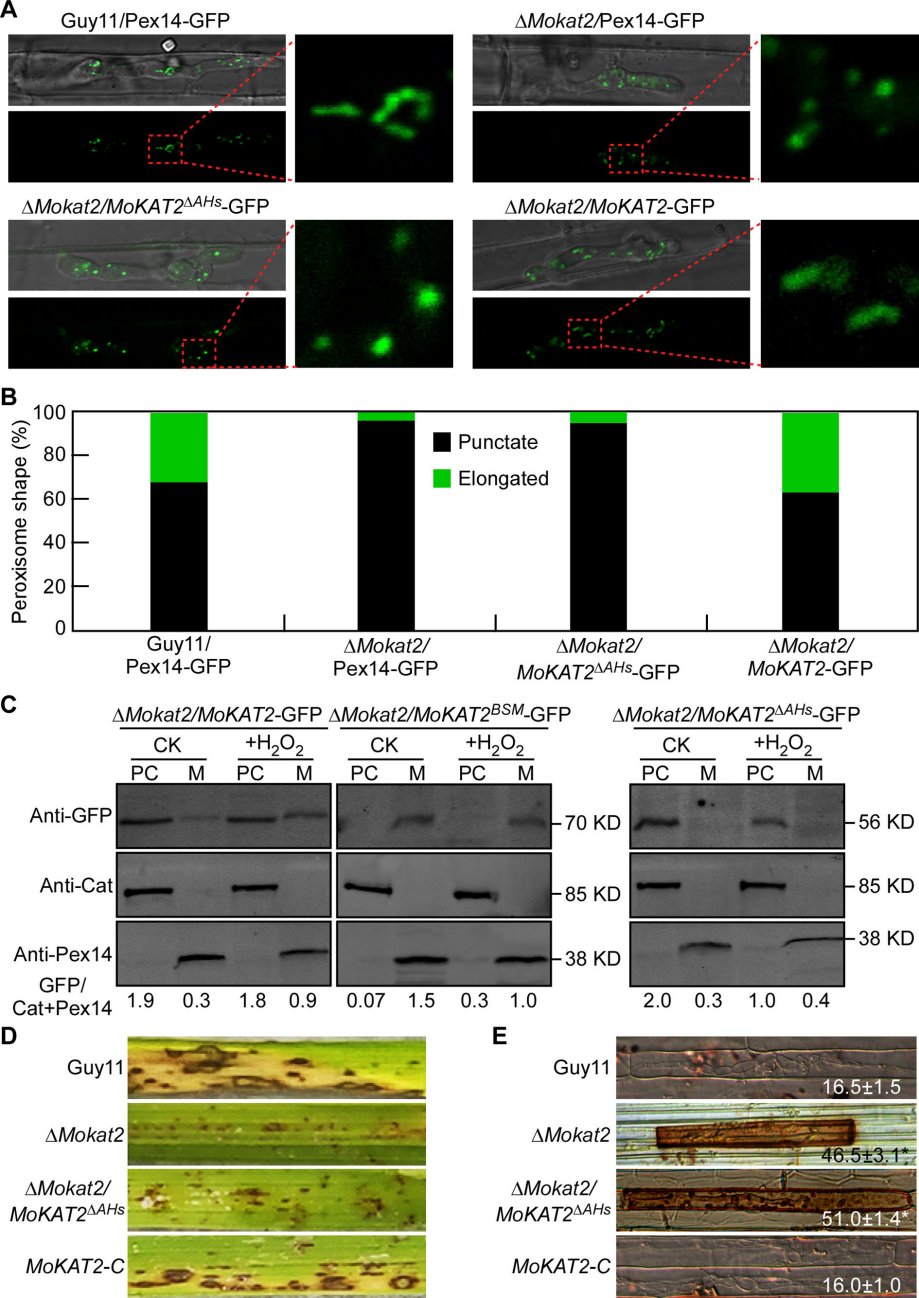
The AH structures of MoKat2 are required for peroxisome elongation in response to oxidative stress. (A) Conidial suspensions of Guy11/Pex14-GFP, Δ*Mokat2*/Pex14-GFP, Δ*Mokat2/MoKAT2*^Δ^*^AHs^*-GFP, and Δ*Mokat2/MoKAT2*-GFP were injected into detached rice sheaths, and peroxisome morphology in IH was examined at 24 hpi, respectively. (B) Statistical analysis of the percentage of peroxisome shape in IH. (C) Western blot analysis of the abundance of MoKat2, MoKat^BSM^, and MoKat^Δ^*^AHs^* in peroxisome cytoplasm (PC) and membrane (M) with or without H_2_O_2_ treatment using anti-GFP and anti-Pex14. Anti-cat antibody was used as a control for detecting peroxisome cytoplasm protein. The relative quantification of MoKat2 accumulated in peroxisome membrane was estimated by calculating the amount of MoKat2-GFP compared with the total amount of intact cat and Pex14 (GFP/cat + Pex14), and the gray values are analyzed by ImageJ. (D) Spraying assay of the indicated strains on rice seedlings. Photographs were taken at 7 dai. (E) Rice sheath cells infected by the indicated strains were stained by DAB at 36 hpi, and examined under a confocal microscope. The number represents the percentage of DAB staining cells. ±SD was calculated from three independent experiments, and asterisk indicates significant differences at *P* < 0.01.

The AH is able to bind to membranes and induce membrane curvature which is crucial for organelle membrane remodeling (48). MoKat2 contains two conserved AHs (Nα3 and Cα3) completely buried in the dimer and sandwiched between two β-sheets similar to ScPot1 (Fig. 2C). Therefore, we hypothesized that MoKat2 exposed AHs by dissociating under oxidative conditions, and accumulated in peroxisome membrane through AHs to regulate membrane curvature, thus controlling peroxisome elongation. To test this possibility, we generated the deletion variant of MoKat2 with the AH sequences deleted (*MoKAT2*^Δ^*^AHs^*) in the Δ*Mokat2* mutant background and examined the peroxisome morphology in IH of Δ*Mokat2/MoKAT2*^Δ^*^AHs^*-GFP transformant. The results showed that 2.3% peroxisomes in IH of Δ*Mokat2/MoKAT2*^Δ^*^AHs^*-GFP exhibited tubular shape and 97.2% with punctate shape at 24 hpi, similar to that found in the Δ*Mokat2/*Pex14-GFP mutant (Fig. 8A and B). We then analyzed the abundance of MoKat2, MoKat2^Δ^*^AHs^*, and MoKat2^BSM^ in peroxisome membrane with or without H_2_O_2_ treatment. The results revealed that more MoKat2 protein was accumulated in peroxisome membrane of Δ*Mokat2/MoKAT2*-GFP when treated with H_2_O_2_ than the untreated control (Fig. 8C, left panel). High abundance of MoKat2^BSM^ protein was detected in peroxisome membrane of Δ*Mokat2/MoKAT2^BSM^*-GFP under both control and H_2_O_2_ conditions, and almost no protein was detected in peroxisome cytoplasm (Fig. 8C, middle panel). In contrast, almost no MoKat2^Δ^*^AHs^* protein was detected in peroxisome membrane of Δ*Mokat2/MoKAT2*^Δ^*^AHs^*-GFP under both control and H_2_O_2_ conditions (Fig. 8C, right panel). We further tested the pathogenicity of Δ*Mokat2/MoKAT2*^Δ^*^AHs^*-GFP and found that it showed reduced virulence similar to the Δ*Mokat2* mutant (Fig. 8D). DAB staining assay also showed similar results to the mutant (Fig. 8E). These findings indicated that AH sequences of MoKat2 are essential for peroxisome elongation via anchoring peroxisome membrane in response to oxidative stress, which are also required for infectious growth and host ROS accumulation of *M. oryzae*.

### MoKat2 thiolase activity does not contribute to peroxisome elongation and host ROS accumulation

**Fig 9:**
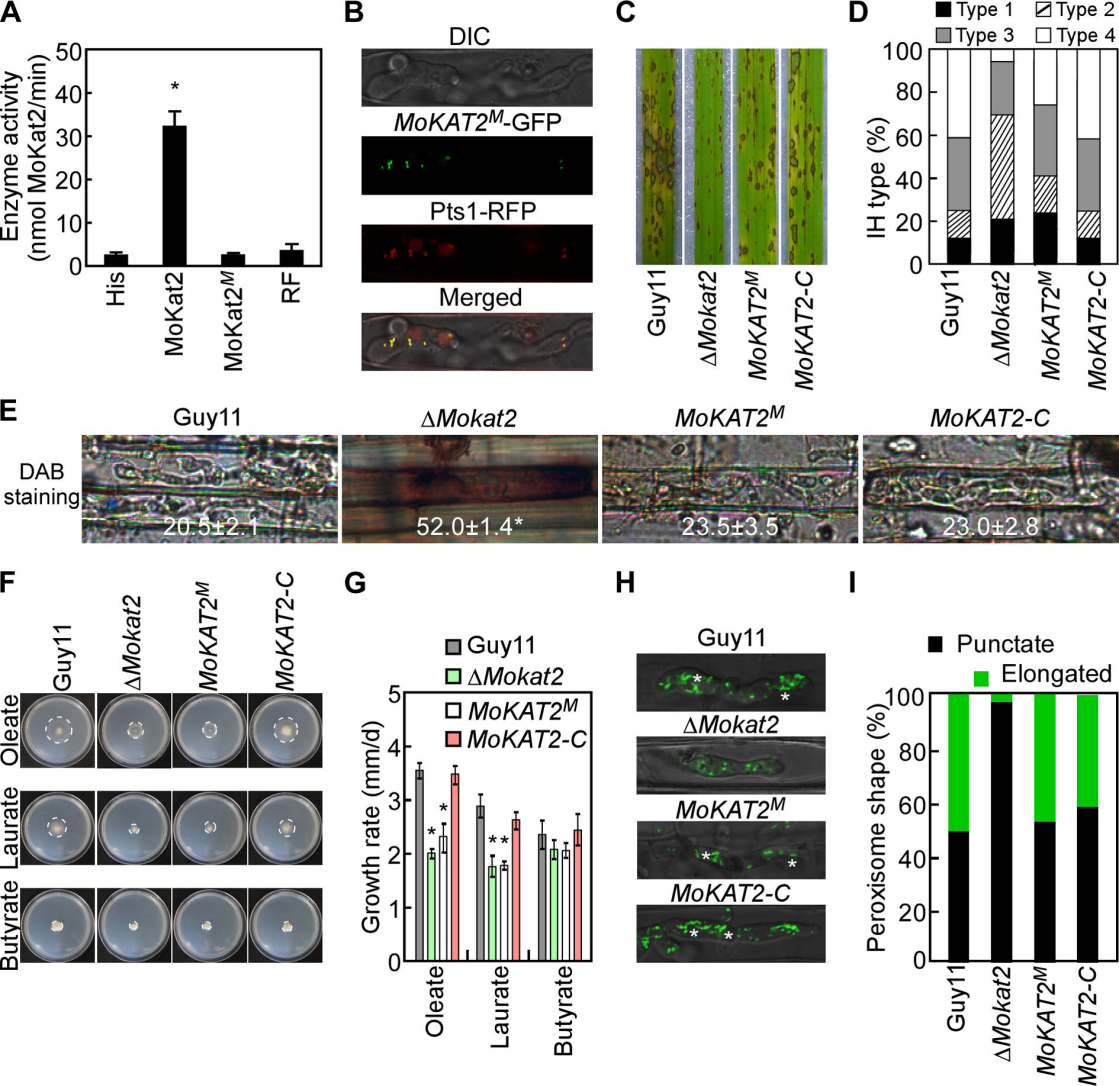
MoKat2 thiolase activity does not contribute to peroxisome elongation and host ROS accumulation. (A) Analysis of the MoKat2^M^ (catalytic sites mutation protein) enzyme activity. His and reagent F (RF) were used as negative control. Error bars are standard deviations from three biological repeats, and asterisk indicates significant differences at *P* < 0.01. (B) Localization pattern of MoKat2^M^ in IH. (C) Spraying assay of indicated strains on rice seedlings. (D) IH-type examination of the indicated strains on rice sheaths. (E) DAB staining of the indicated strains and statistical analysis of the percentage of the rice cells stained by DAB. (F) The indicated strains were inoculated onto MM medium with oleate, laurate, and butyrate as sole carbon sources, respectively, and photographed at 7 dpi. (G) The growth rate was calculated by diameter/days. Error bars are standard deviations from three biological repeats, and asterisks indicate significant differences at *P* < 0.01. (H) Examination of the peroxisome morphology in IH at 24 hpi. White asterisks indicate the elongated peroxisomes. (I) Statistical analysis of the percentage of different peroxisome shapes in IH.

To further clarify the role of MoKat2, we generated its putative thiolase catalytic sites (C119, H375, and C405) (46) mutation transformant MoKat2^M^ in the Δ*Mokat2* mutant background. *In vitro* enzymatic assay showed very low thiolase activity of MoKat2^M^ compared to MoKat2 (Fig. 9A). Subcellular localization assay showed a peroxisome location of MoKat2^M^ in IH (Fig. 9B). Pathogenicity assay revealed a partial recovery of infectious growth of MoKat2^M^ compared to the Δ*Mokat2* mutant (Fig. 9C and D). DAB staining assay exhibited similar results of MoKat2^M^ to Guy11 and *MoKAT2-C* which with much lower rice cells stained by DAB compared to Δ*Mokat2* (Fig. 9E). Fatty acid oxidation assay showed a lower growth rate of MoKat2^M^ on oleate and laurate medium, similar to Δ*Mokat2* (Fig. 9F and G). Appressorium turgor assay revealed a decreased turgor of MoKat2^M^ that is similar to Δ*Mokat2* (Table S2-2). We finally examined the peroxisome morphology of MoKat2^M^ in IH at 24 hpi and found that the percentage of elongated peroxisome of MoKat2^M^ was very close to Guy11 and *MoKAT2-C* (Fig. 9H and I). These results suggested that MoKat2 thiolase activity does not contribute to peroxisome elongation and host ROS accumulation, but participates in fatty acid oxidation.

## DISCUSSION

**Fig 10:**
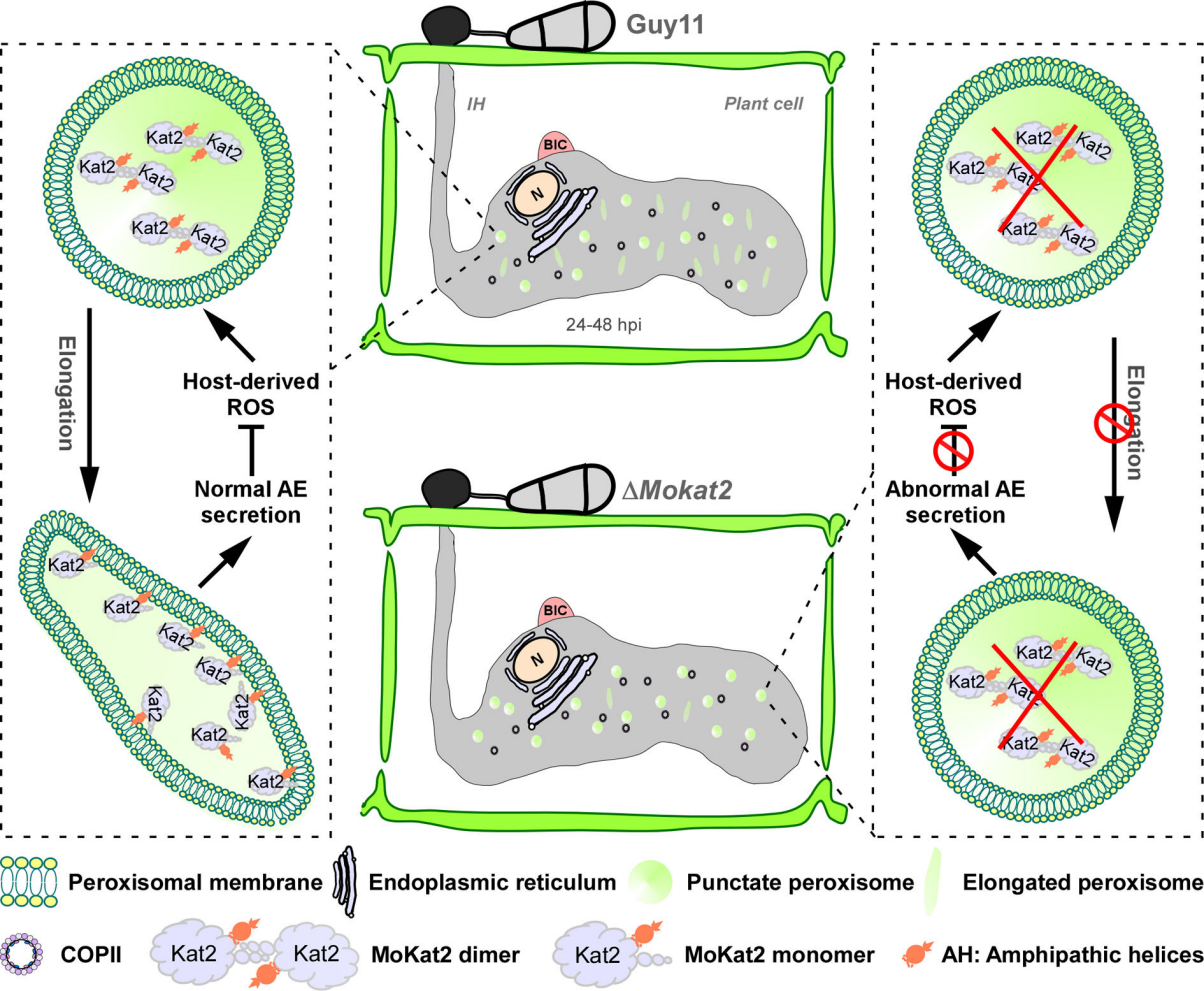
The putative regulatory mechanism of peroxisome dynamics during early infection of *M. oryzae* mediated by MoKat2 in response to host-derived ROS. AE: apoplastic effector.

In this study, we delve into the fascinating realm of peroxisome dynamics in the rice blast fungus *M. oryzae* during its infection. Our findings reveal an intriguing interplay between the host and pathogen, as the peroxisome undergoes dynamic configuration changes during infection. Our research further unveils that peroxisome elongation is contingent upon host-derived ROS, and this alteration is directly linked to the accumulation of ROS within the host and the infectious growth of *M. oryzae*. Additionally, we successfully identify a key player MoKat2 in this process, which is likely responsible for peroxisome elongation in response to host-derived ROS. Our investigation uncover that MoKat2 dimer undergoes dissociation as a result of host produced ROS during early infection and demonstrate that the binding of MoKat2 monomer to peroxisome membranes via its AHs is imperative for its involvement in the aforementioned processes. We further demonstrate that MoKat2 modulates the proper secretion of the apoplastic effectors to suppress host ROS generation, thus facilitating the infectious growth of *M. oryzae* (Fig. 10). Our findings shed light on the intricate relationship between MoKat2 and peroxisome dynamics, illuminating their crucial role in the accumulation of host-derived ROS and the successful infection of *M. oryzae*. This represents a major step forward in understanding how phytopathogens utilize peroxisome to counteract host-derived ROS during pathogen-host interactions.

In signaling and metabolic networks, changes in metabolites, signal molecules, and gene expression have been widely studied while the role of the dynamic properties and crosstalk of organelles in response to the environment have been less explored (33). Organelle dynamics is necessary for cell functionality, including crosstalk between organelles, and for signaling (49). Peroxisome is highly dynamic and metabolically active and is known as a sensitive oxidative stress response organelle that is extremely dynamic with regard to their shape, distribution, and movement (50, 51). Here, we found that peroxisome undergoes dynamic changes during infection dependent on host-derived ROS, and its elongation determines the host ROS accumulation and infectious growth, indicating that peroxisome is a key organelle in response to ROS in phytopathogens. Despite any ROS receptor needing to be characterized, pathogen contains several conserved MAP kinase pathways, including MoOsm1 pathway of *M. oryzae* in signal transduction under stress conditions (8, 52). We, here, identified that MoKat2 likely acts as a potential ROS sensor to combat oxidative stress by regulating peroxisome morphology. MoKat2 dimer is dissociated under oxidative conditions and during early infection stage. Future studies are needed to explore how it is dissociated by host-derived ROS *in vivo*. In pathogens, secretion of extracellular catalase-peroxidases to eliminate host-derived ROS and secretion of effectors to suppress host ROS generation are considered as two major strategies to overcome host immunity. The activity of catalase and superoxide dismutase was not affected in the Δ*Mokat2* mutant, but the expression of one of the genes responsible for rice ROS generation was significantly increased in the mutant, indicating MoKat2 is able to suppress host basal immunity. The abnormal secretion of the apoplastic effectors in the Δ*Mokat2* mutant further supports this finding. However, the underlying mechanisms of how MoKat2 modulates the secretion of these effectors need to be further investigated.

AHs bind lipids and are involved in vesicular transport and membrane modification (48). Many cytosolic proteins have been found to interact in a reversible manner with membrane-bound organelles through AHs. Studies on proteins involved in vesicular transport have revealed that some AHs do not act as simple membrane anchors but instead as tools to deform lipid membranes or sense membrane curvature (48). For example, AH regions of ArfGAP1, which are conserved from yeast to mammals, act as a lipid-packing sensor and help anchor ArfGAP1 at the surface of highly curved membrane, thus allowing GTP hydrolysis on Arf1 (53). The mechanisms used to induce membrane curvature may allow molecules to sense its presence. Thus, certain proteins are able to upconcentrate on areas of high membrane curvature, such as Bin/Amphiphysin/Rvs domains and AHs (54, 55). Because membrane curvature cannot be measured quantitatively *in vivo*, so far all membrane curvature-dependent interactions have been characterized *in vitro* (56). In addition, previous studies showed that AHs fold upon contacting membranes and insert their hydrophobic face in the lipid bilayer often with the help of positively charged residues situated on the polar face of the helix (57). In our study, the abundance of MoKat2 in peroxisome membrane is higher in the monomer and decreases in the AHs deletion mutant. Combined with the peroxisome morphology of the AHs mutant, we speculate that the AHs of MoKat2 affect peroxisome elongation possibly through regulating peroxisome membrane curvature. However, the direct evidence of how AHs affect peroxisome membrane curvature needs to be further explored.

Since MoKat2 is a 3-ketoacyl-CoA thiolase which catalyses a key step in fatty acid β*-*oxidation, it is reasonable that MoKat2 is involved in long-chain and medium-chain fatty acid oxidation. Interestingly, constitutive MoKat2 monomer shows a higher ability to oxidize long-chain fatty acid than the wild type, as well as a faster extension of infectious growth. We hypothesize that during early infection phases, *M. oryzae* must quickly overcome host immunity by eliminating host-derived ROS on one hand, and on the other hand, it needs to quickly activate the fatty acids metabolism pathway in IH to promote its infection and colonization. This is because, during biotrophic infection stage, rice cell is a relative nutrient starvation condition with non-preferred carbon sources such as fatty acids (58). MoKat2 is a perfect candidate for *M. oryzae* to involve in these two key processes by regulating peroxisome elongation in response to host-derived ROS. One possible explanation is that the elongated peroxisomes increase the surface-to-volume ratio of the organelle which facilitates proteins and/or metabolite export, likely the secretion of extracellular proteins. For the increased fatty acid oxidation activity of the MoKat2 monomer, the possibility is that the elongated peroxisome promotes the physical interaction between peroxisome and LD (source of fatty acids) and accelerates the LD oxidation to supply the energy for the IH to overcome host immunity and IH extension. Similar findings were reported in mammals that Spastin promotes peroxisome-LD contact formation to facilitate LD peroxidation (59). Additionally, we found that the thiolase activity of MoKat2 is not involved in peroxisome elongation and host ROS accumulation, but is important for fatty acid oxidation, indicating the multiple roles and complex regulatory mechanism of MoKat2. Previous study has shown that over-expression of 3-ketoacyl-CoA thiolase resulted in loss of pathogenicity of *Leptosphaeria maculans* (60). These findings implied that the metabolic balance of the pathogenic fungi is critical for their normal function during pathogen-plant interactions. Nevertheless, the in-depth regulatory mechanism of MoKat2 on fatty acid oxidation also needs to be further studied.

## MATERIALS AND METHODS

### Strains and culture conditions

*M. oryzae* Guy11 was used as the wild-type strain in this study. For vegetative growth, 2 mm × 2 mm mycelial blocks were placed onto freshly prepared complete medium (CM), MM, or MM with 1% (wt/vol) glucose, 1 mM sodium butyrate, sodium laurate, and sodium oleate as sole carbon sources (61). Two-day-old liquid CM cultures were used for DNA and RNA extraction. For conidiation, mycelial blocks were inoculated onto straw decoction and corn agar medium at 28°C for 7 days in the dark and followed by 3 days of continuous illumination under fluorescent light (62).

### Targeted gene deletion and complementation

To generate gene deletion construct, two fragments with 1.0 kb of sequence flanking the targeted gene were amplified with primer pairs and ligated to the hygromycin resistance cassette (*HPH*) released from pCX62. The resulting constructs were transformed into the protoplasts of the wild-type Guy11 using polyethylene glycol-mediated approach. To generate complementation transformants, fragments containing the entire *MoKAT2* gene coding region and a 1.5 kb sequence upstream of its start codon were amplified and inserted into pYF11 (bleomycin resistance) fusing with GFP. The resulting pYF11-*MoKAT2*-GFP constructs were sequenced and re-introduced into protoplasts of the Δ*Mokat2* mutant. The complemented transformants *MoKAT2*-C were screened by GFP signal and confirmed by the recovered phenotypes. Primers used in this study are listed in Table S3.

### ROS observation

Rice sheaths were inoculated with conidial suspensions of the indicated strains for 36 h, and then incubated in 1 mg/mL DAB solution (Sigma-Aldrich), pH 3.8, at 25°C for 8 h and destained with clearing solution (ethanol:acetic acid, 94:4 [vol/vol]) for 1 h. The epidermises of infected cells were observed under a microscope. In addition, molecular probes CM-H2DCFDA (Thermo Fisher Scientific, C6827) (63) offer derivatives of reduced fluorescein and calcein as cell-permeant indicators for ROS. Rice sheaths were inoculated with the mutant and wild-type strains for 36 hpi, then the epidermises of infected cells were incubated in 5 µM CM-H2DCFDA for 5 min in the dark and washed with 1× phosphate buffered saline (PBS) for three times, and observed under a microscope.

### Transcriptome sequencing and functional analysis

Total RNA was obtained from control and treated samples by an Easy Pure RNA Kit (TransGen, Biotech, China). In brief, cultures grown in complete medium (CM) with 5 mM H_2_O_2_ treatment for 20 min after 48 h were used as the treatment group (H_2_O_2__1, H_2_O_2__2). Simultaneously, cultures grown in CM without H_2_O_2_ treatment 1 were used as the control group (CK_1, CK_2). The infected leaves at 24 hpi were used as the treatment group 2 (IH24h_1, IH24h_2). RNA extraction, mRNA transcription, and cDNA library construction were performed by Personal Biotechnology Co. (Shanghai, China). The cDNA library was sequenced on the Illumina HiSeq 2500 platform (64). *De novo* transcriptome assembly was conducted, and the unigenes were annotated using the Gene Ontology database and KEGG to analyze the DEGs. The BLAST algorithm (blastx/blastp) was used to identify the potential biological pathways of the genes.

### qRT-PCR analysis

Total RNA was reverse transcribed into first-strand cDNA using the oligo (dT) primer and HiScript II Q select RT SuperMix for qPCR (Vazyme, Nanjing, R233-01). The qRT-PCR was run on the Applied Biosystems 7500 Real-Time PCR System with ChamQ SYBR qPCR Master Mix (Vazyme, Nanjing, Q311-02). Normalization and comparison of mean Ct values were performed as described previously (8).

### Epifluorescence microscopy observation

Vegetative hyphae, conidia, appressoria, and infectious hyphae expressing fluorescent protein-fused target genes were incubated under appropriate conditions. The constructs including MoPex14-GFP, MoKat2-GFP, MoPts1-RFP, and other mutation vectors were transformed into Δ*Mokat2* mutant or the wild-type Guy11 strain. Epifluorescence microscopy was performed using a Zeiss LSM710 (63× oil) microscope.

### Yeast two-hybrid assays

Bait constructs were conducted by cloning full-length cDNAs of target genes into pGADT7 and pGBKT7 (LMAI Biotechnology, LM1010). Prey constructs and bait constructs were confirmed by DNA sequencing and co-transformed into the yeast strain AH109 (LMAI Biotechnology, LM1010) following the recommended protocol (BD Biosciences Clontech). Transformants screened by synthetic dextrose medium minus leucine, tryptophan, and histidine (SD-Leu-Trp-His) were selected. Yeast strains for positive and negative controls were provided by the BD library construction and screening kit (65).

### Co-IP assay

MoKat2-GFP, MoKat2-FLAG, and all of the mutation protein fusion constructs were constructed by yeast homologous recombination transformation. Different pairs of specific constructs were co-transformed into the protoplasts of the wild-type strain. Total proteins were isolated from the positive transformants and incubated with anti-FLAG agarose (Chromo Tek, gta-20) at 4°C for 4 h with gently shaking. Proteins bound to the beads were eluted after a series of washing steps by 1× PBS. Elution buffer (200 mM glycine, pH 2.5) and neutralization buffer (1 M Tris, pH 10.4) were used for the elution process. Total, suspension, and eluted proteins were analyzed by western blot using FLAG (mouse, 1:5,000; Abmart, M20018) or GFP (mouse, 1:5,000; Abmart, 293967) specific antibodies.

### BiFC assays

The protein MoKat2 fusion constructs were generated by cloning the full length of *MoKAT2* with 1,500 bp native promoter region into pHZ65 and pHZ68, respectively (66). The resulting constructs of MoKat2-YFP^N^ and MoKat2-YFP^C^ were co-introduced into the protoplasts of Δ*Mokat2* mutant. Transformants resistant to both hygromycin (Solarbio Life Sciences, H8080) and zeocin (Thermo Fisher Scientific, R25001) were isolated and confirmed by PCR and yellow fluorescent protein (YFP) signals.

### Blue native gel electrophoresis

For total protein extraction, strains were cultured in liquid CM media with shaking for 36 h, and then treated with or without 5 mM H_2_O_2_ for 30 min. Mycelia were collected and ground into a fine powder, then resuspended in 1× NativePAGE (NativePAGE Bis-Tris Gel System, Thermo Fisher Scientific, BN1002BOX) sample buffer (100 mM Tris-Cl [pH 8.0], 40% glycerol, 0.5% Coomassie brilliant blue G-250) and incubated for 5 min at room temperature. Protein samples were loaded onto 4%–16% discontinuous blue native polyacrylamide gels and separated at 4°C in cathode buffer containing 100 mM Histidine (pH 8.0) and 0.002% Coomassie brilliant blue G-250. The gels were fixed and detained in 7.5% acetic acid and 5% ethanol (67).

### Protein extraction and western blot analysis

For total protein extraction, mycelia were cultured in liquid CM media with shaking for 36 h, and then treated with or without 5 mM H_2_O_2_ for 30 min. Subsequently, mycelia were ground into a fine powder in liquid nitrogen and resuspended in 1 mL lysis buffer (10 mM Tris-HCl, pH 7.5, 150 mM NaCl, 0.5 mM EDTA, 0.5% NP-40) with 2 mM phenylmethanesulfonyl fluoride (PMSF) and proteinase inhibitor cocktail. The lysates were placed on ice for 30 min and shaken for three times. Cell debris was removed by centrifugation at 13,000*g* for 10 min at 4°C. The lysates were collected as total proteins. For GFP-tagged protein detection, samples were separated by 10% SDS-PAGE and followed by western blot analysis.

The membrane and cytosol fractions were extracted using a membrane and cytosol protein extraction kit (Beyotime, P0033). The treated mycelia were ground in liquid nitrogen and added to a centrifuge tube with 1 mL of buffer A containing 1 mM PMSF, shaken on a vortex intensely for 30 s, and then centrifuged at 700*g* for 10 min at 4°C. The resulting supernatant was transferred to a new centrifuge tube without any precipitation and was centrifuged at 14,000*g* for 10 s at 4°C. The supernatant was transferred to a new centrifuge tube (cytosol fraction), and 200 µL of buffer B was added to the precipitate. The sample was shaken on a vortex intensely for 10 s and kept on ice for 10 min. This shaking and ice step was repeated twice and then centrifuged at 14,000*g* for 10 min at 4°C to recover the supernatant’s membrane fraction (68). Proteins from the sample lysate were fractionated using 10% SDS-PAGE. Blot signals were detected and analyzed using the Odyssey infrared imaging system (version 2.1) as described previously (8).

### Statistical analysis

Mean and standard deviation (SD) were calculated from at least three independent replicates. The significance of differences between samples was statistically evaluated using SD and analysis of variance in GraphPad Prism 8.0.1 as described previously (58).

## Data Availability

Genes reported in this study are able to be found in the GenBank database (https://www.ncbi.nlm.nih.gov/protein/) using the following accession numbers: MGG_09512, XP_003712230.1XP_003712230.1; MGG_13647, XP_003717586.1; MGG_10700, XP_003720344.1; MGG_06561, XP_003716979.1; MGG_04956, XP_003712452.1; MGG_17054, XP_003716242.1; MGG_06148, XP_003712026.1; MGG_06332, XP_003717250.1.

## SUPPLEMENTAL MATERIAL

Fig. S1 mbio.02381-23-s0001.docx *M. oryzae* peroxisome undergoes dynamic changes during infection.

Fig. S2 mbio.02381-23-s0002.docx Transcriptional data, DEG identification, and KEGG pathway enrichment analysis of the differential genes.

Fig. S3 mbio.02381-23-s0003.docx Targeted deletion of MoKAT2 in *M. oryzae*.

Fig. S4 mbio.02381-23-s0004.docx MoKat2 is involved in lipid droplet and glycogen degradation during appressorium development.

Fig. S5 mbio.02381-23-s0005.docx MoKat2 has no role in regulating superoxide dismutase (SOD) and catalase (CAT) activity, and the secretion of the cytoplasmic effectors.

Fig. S6 mbio.02381-23-s0006.docx MoKat2 is responsible for peroxisome elongation in response to extracellular oxidants.

Fig. S7 mbio.02381-23-s0007.docx MoKat2 is dissociated under exogenous H_2_O_2_ treatment.

Table S1 mbio.02381-23-s0008.docx Summary of the sequencing and assembly results, and data filtering of the sequencing results.

Table S2 mbio.02381-23-s0009.docx Prediction of the protein subcellular localization and phenotypic analysis of Guy11, ∆Mokat2, MoKAT2-C, and MoKAT2M strains.

Table S3 mbio.02381-23-s0010.docx Primers used in this study.

ASM does not own the copyrights to Supplemental Material that may be linked to, or accessed through, an article. The authors have granted ASM a non-exclusive, world-wide license to publish the Supplemental Material files. Please contact the corresponding author directly for reuse.
