# Supplementary material for: Peroxisome dynamics determines host-derived ROS accumulation and infectious growth of the rice blast fungus
Source: mBio. 2023 Nov 15;14(6):e02381-23. doi: 10.1128/mbio.02381-23 (PMC10746245; doi:10.1128/mbio.02381-23)
Supplement: Fig. S1 — M. oryzae peroxisome undergoes dynamic changes during infection. [file mbio.02381-23-s0001.docx]

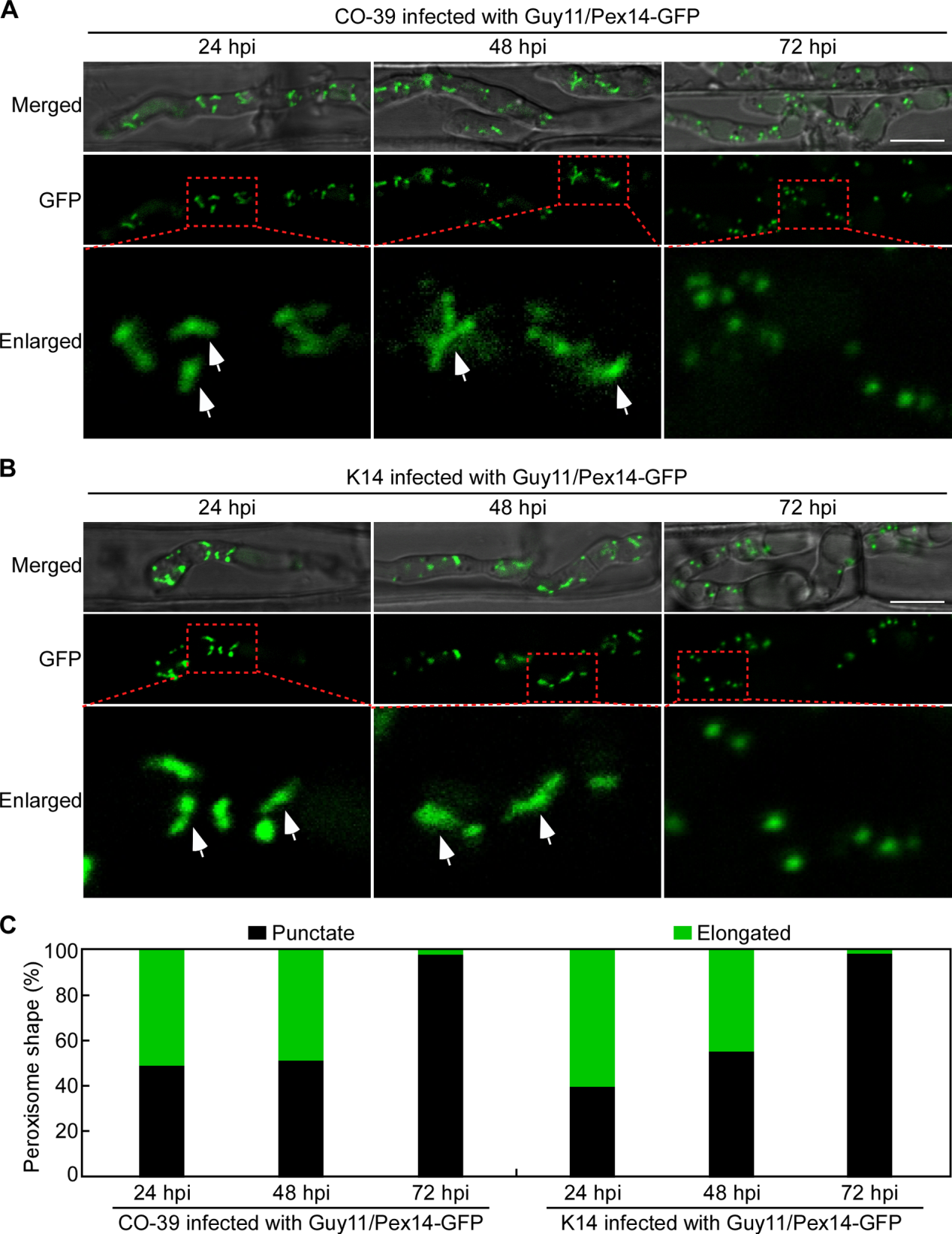


**Figure S1. *M. oryzae* peroxisome undergoes dynamic changes during infection.** (A) Conidial suspensions of Guy11 expressing Pex14-GFP were injected into detached rice sheaths of cultivar CO-39; and peroxisome morphology in IH were examined at 24, 48 and 72 hpi. Bar=10 μm. White arrows indicate the elongated peroxisomes. (B) Conidial suspensions of Guy11 expressing Pex14-GFP were injected into detached rice sheaths of cultivar K14; and peroxisome morphology in IH were examined at 24, 48 and 72 hpi. Bar=10 μm. (C) Statistical analysis of the percentage of peroxisome shape in IH.
