# Supplementary material for: Peroxisome dynamics determines host-derived ROS accumulation and infectious growth of the rice blast fungus
Source: mBio. 2023 Nov 15;14(6):e02381-23. doi: 10.1128/mbio.02381-23 (PMC10746245; doi:10.1128/mbio.02381-23)
Supplement: Fig. S2 — Transcriptional data, DEG identification, and KEGG pathway enrichment analysis of the differential genes. [file mbio.02381-23-s0002.docx]

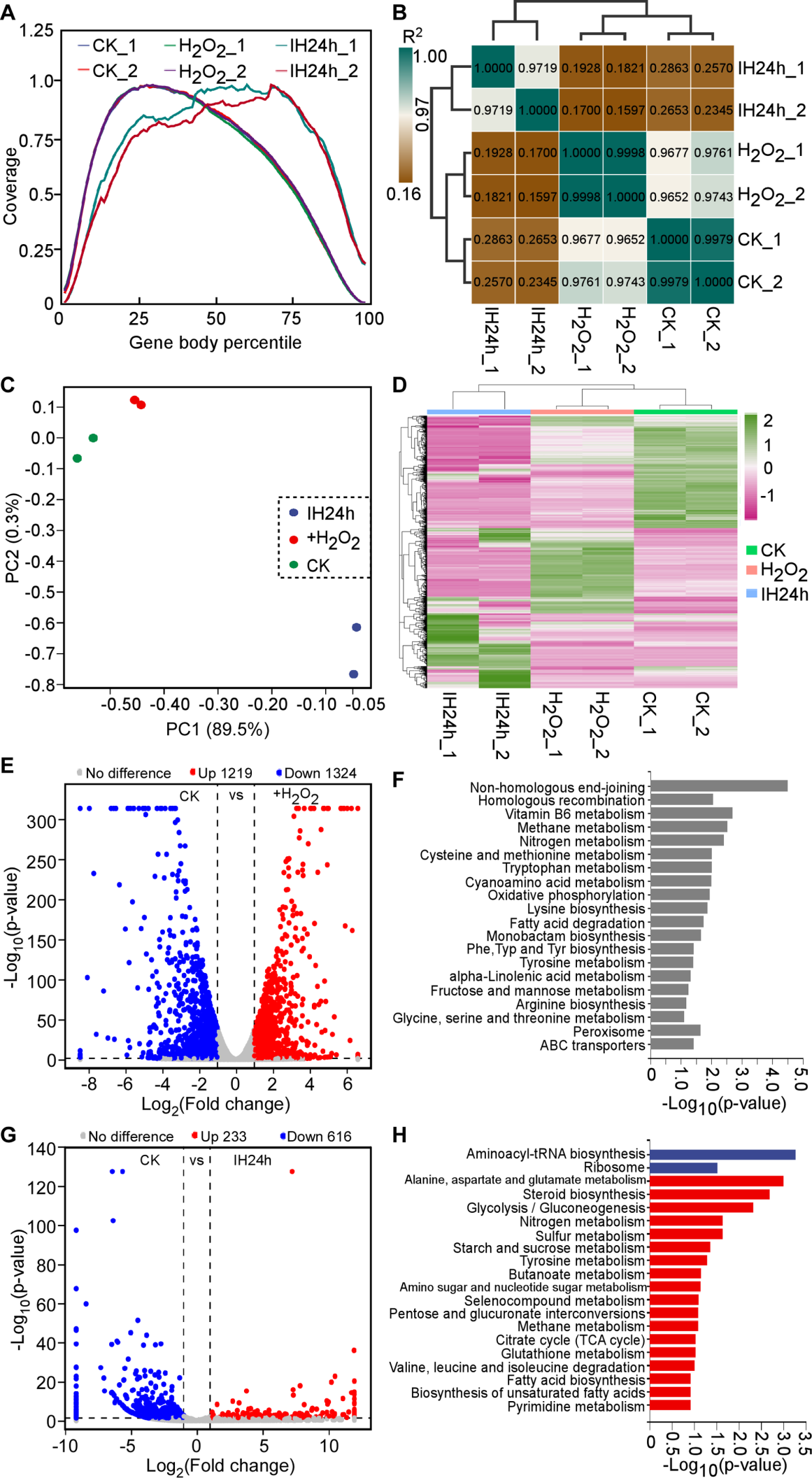


**Figure S2. Comparison of the transcriptional data, and DEG identification, KEGG pathway enrichment analysis of the differential genes.** (A) Average degree of gene coverage. The abscissa is the percentage of the base length of a single gene to the total base length, 0 denotes the 5', 100 denotes the 3'. The ordinate is the sum of the sequence number in the corresponding interval on the horizontal axis of all genes. (B) Sample correlation test. The left and upper sides are the sample clustering, the right and lower sides in the figure are the sample names, and the squares with different colors represent the correlation of the two samples. (C) Principal component analysis (PCA) of the gene changes. (D) Heatmap of DEGs in control group (CK), treatment group (+H_2_O_2_) and invasive hyphae group (IH24h). Horizontal indicates genes; one sample per column, purple indicates highly expressed genes and green indicates low expressed genes. (E) Differential gene volcano plot of +H_2_O_2_ VS CK. Red dots represent up-regulated genes and green dots represent down-regulated genes. (F) KEGG pathway enrichment analysis of the differential genes (+H_2_O_2_ VS CK). (G) Differential gene volcano plot of IH24h VS CK. Red dots represent up-regulated genes and green dots represent down-regulated genes. (H) KEGG pathway enrichment analysis of the differential genes (IH24h VS CK).
