# Supplementary material for: Peroxisome dynamics determines host-derived ROS accumulation and infectious growth of the rice blast fungus
Source: mBio. 2023 Nov 15;14(6):e02381-23. doi: 10.1128/mbio.02381-23 (PMC10746245; doi:10.1128/mbio.02381-23)
Supplement: Fig. S3 — Targeted deletion of MoKAT2 in M. oryzae. [file mbio.02381-23-s0003.docx]

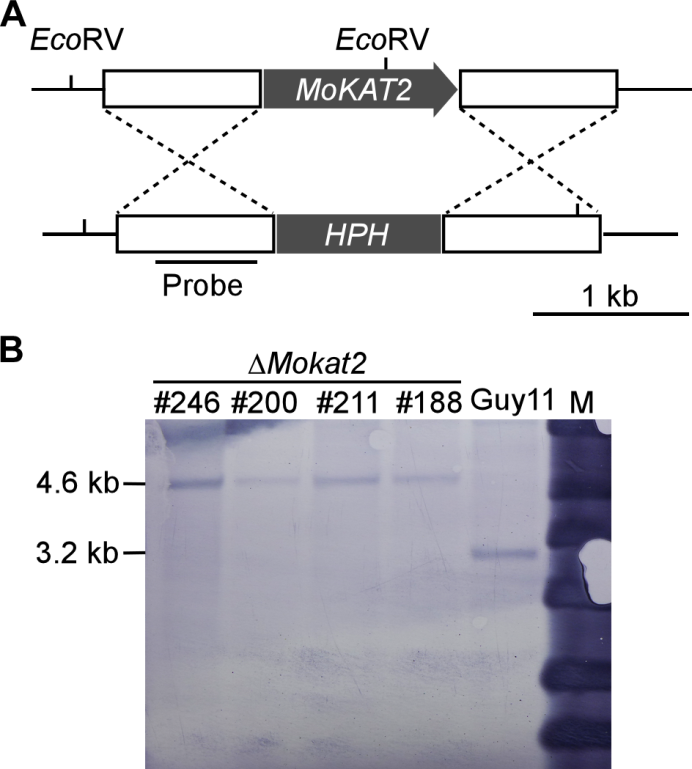


**Figure S3. Targeted deletion of *MoKAT2* in *M. oryzae*.** (A) *MoKAT2* gene replacement strategy in *M. oryzae* genome. (B) *MoKAT2* gene deletion mutants were verified by Southern blot analysis with probe. The genomic DNA was digested with *Eco*RV. M: DNA marker.
