# Supplementary material for: Peroxisome dynamics determines host-derived ROS accumulation and infectious growth of the rice blast fungus
Source: mBio. 2023 Nov 15;14(6):e02381-23. doi: 10.1128/mbio.02381-23 (PMC10746245; doi:10.1128/mbio.02381-23)
Supplement: Fig. S4 — MoKat2 is involved in lipid droplet and glycogen degradation during appressorium development. [file mbio.02381-23-s0004.docx]

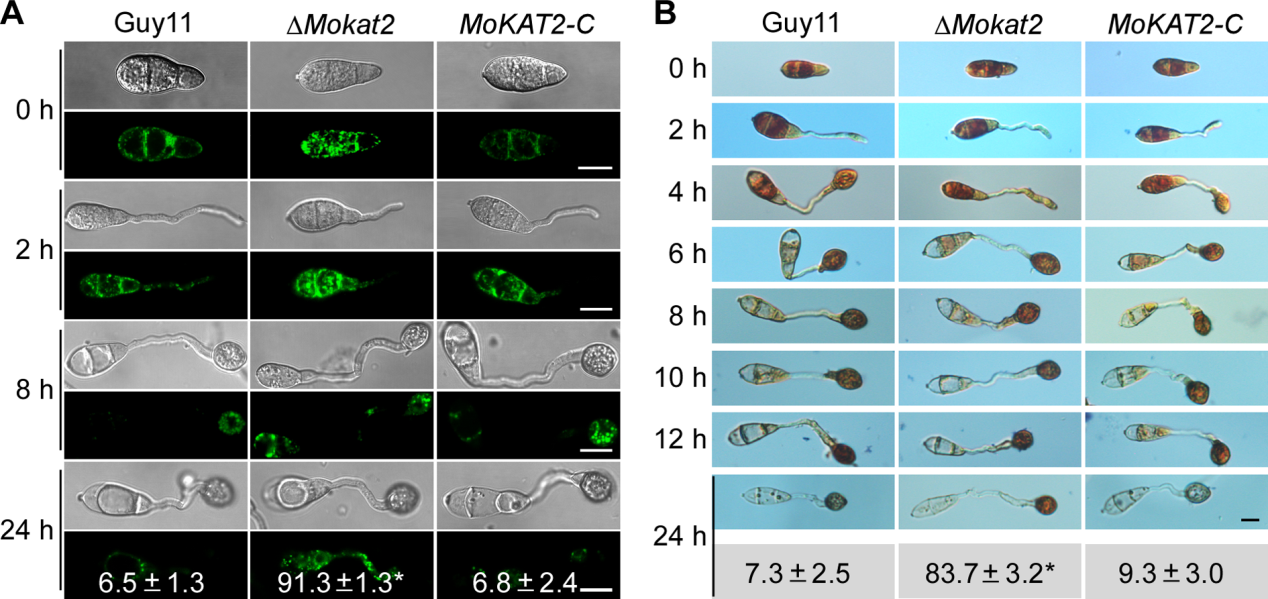


**Figure S4. MoKat2 is involved in lipid droplet and glycogen degradation during appressorium development.** (A) Conidial suspensions of Guy11, Δ*Mokat2* and *MoKAT2-C* were drop inoculated onto a cover glass to induce appressorium formation. Lipid droplets were stained by BODIPY^TM^ 493/503 at different time points and examined under a confocal microscope. Bar=10 μm. (B) Glycogen in conidia and appressoria were stained by iodine solution and examined under a confocal microscope. Bar=10 μm. The numbers indicate the percentage of appressoria containing glycogen at 24 h. ±SD was calculated from three independent experiments, and asterisk indicates significant difference at *p*<0.01.
