# Supplementary material for: Peroxisome dynamics determines host-derived ROS accumulation and infectious growth of the rice blast fungus
Source: mBio. 2023 Nov 15;14(6):e02381-23. doi: 10.1128/mbio.02381-23 (PMC10746245; doi:10.1128/mbio.02381-23)
Supplement: Fig. S5 — MoKat2 has no role in regulating superoxide dismutase (SOD) and catalase (CAT) activity, and the secretion of the cytoplasmic effectors. [file mbio.02381-23-s0005.docx]

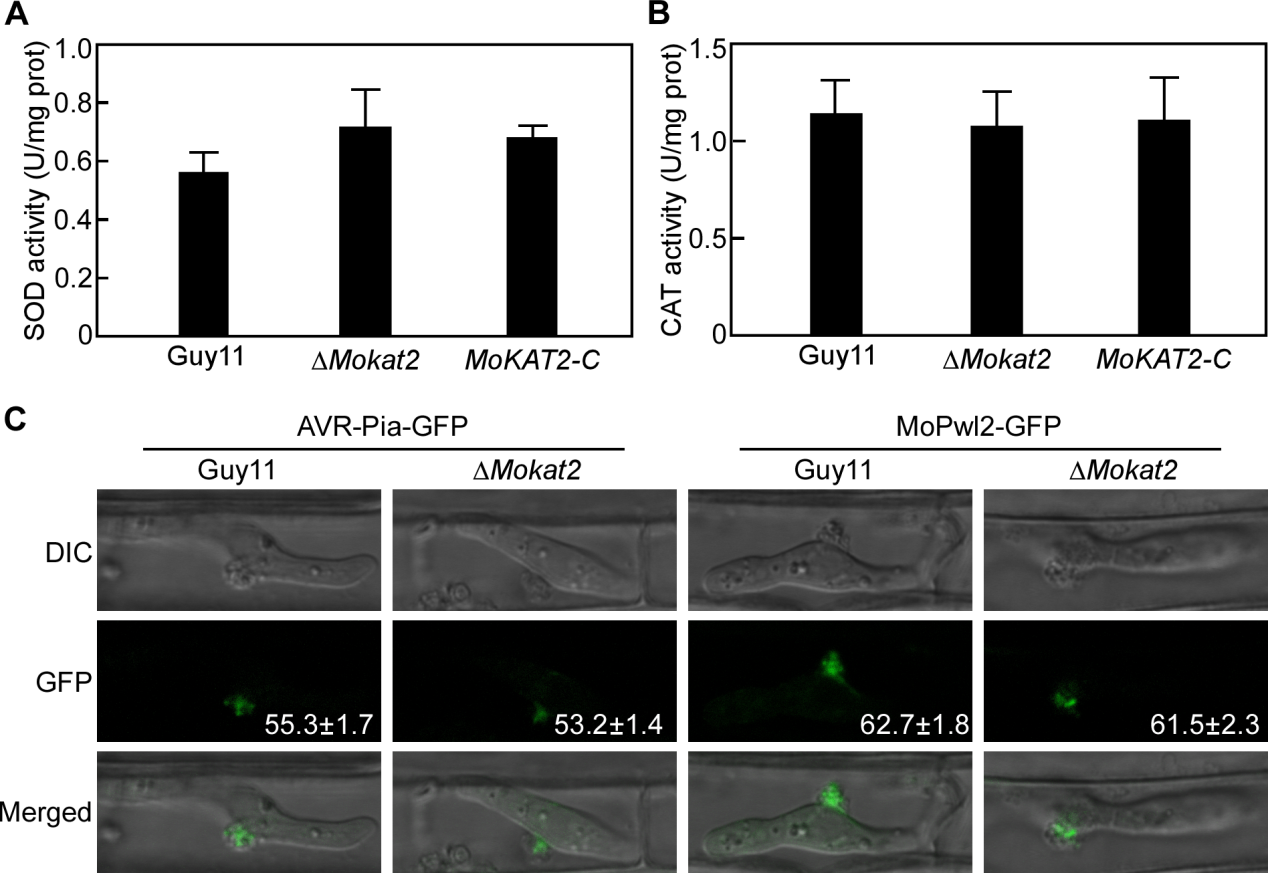


**Figure S5. MoKat2 has no role in regulating superoxide dismutase (SOD) and catalase (CAT) activity, and the secretion of the cytoplasmic effectors.** (A and B) Micromethod assay analyses SOD and CAT activity in Guy11, Δ*Mokat2* and *MoKAT2-C* strains using SOD Activity Assay Kit and CAT Activity Assay Kit (NO. D799598, Sangon Biotech). Error bars are standard deviations from three biological repeats. (C) Conidial suspensions of Guy11 and Δ*Mokat2* separately expressing MoAVR-Pia-GFP and MoPwl2-GFP were injected into detached rice sheaths of cultivar CO-39; and the secretion of the indicated proteins were examined at 24 hpi under a fluorescence microscope. The number indicated the percentage of the localization pattern of the corresponding image. Bar=10 μm.
