# Supplementary material for: Peroxisome dynamics determines host-derived ROS accumulation and infectious growth of the rice blast fungus
Source: mBio. 2023 Nov 15;14(6):e02381-23. doi: 10.1128/mbio.02381-23 (PMC10746245; doi:10.1128/mbio.02381-23)
Supplement: Fig. S6 — MoKat2 is responsible for peroxisome elongation in response to extracellular oxidants. [file mbio.02381-23-s0006.docx]

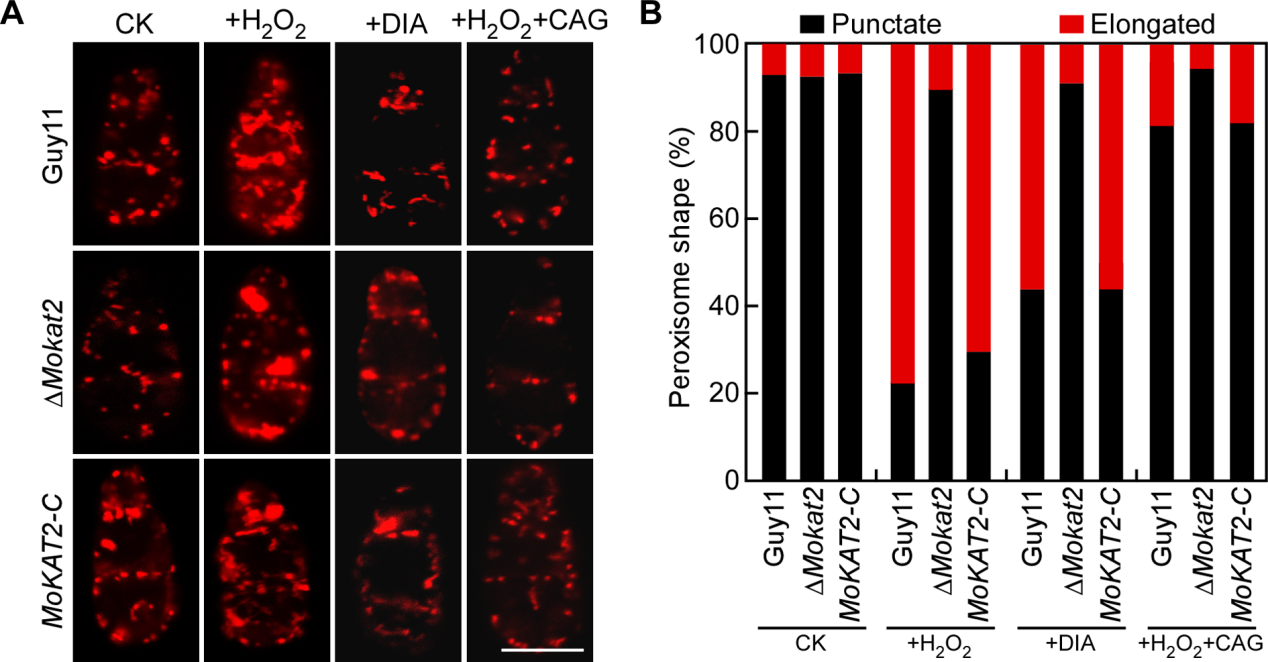


**Figure S6. MoKat2 is responsible for peroxisome elongation in response to extracellular oxidants.** (A) Conidia of Guy11, Δ*Mokat2* and *MoKAT2-C* expressing Pts1-RFP were treated with H_2_O_2_ or DIA, and peroxisome morphology was examined under a confocal microscope. Bar=10 μm. (B) Statistical analysis of the percentage of peroxisome shape in conidia.
