# Supplementary material for: Peroxisome dynamics determines host-derived ROS accumulation and infectious growth of the rice blast fungus
Source: mBio. 2023 Nov 15;14(6):e02381-23. doi: 10.1128/mbio.02381-23 (PMC10746245; doi:10.1128/mbio.02381-23)
Supplement: Fig. S7 — MoKat2 is dissociated under exogenous H2O2 treatment. [file mbio.02381-23-s0007.docx]

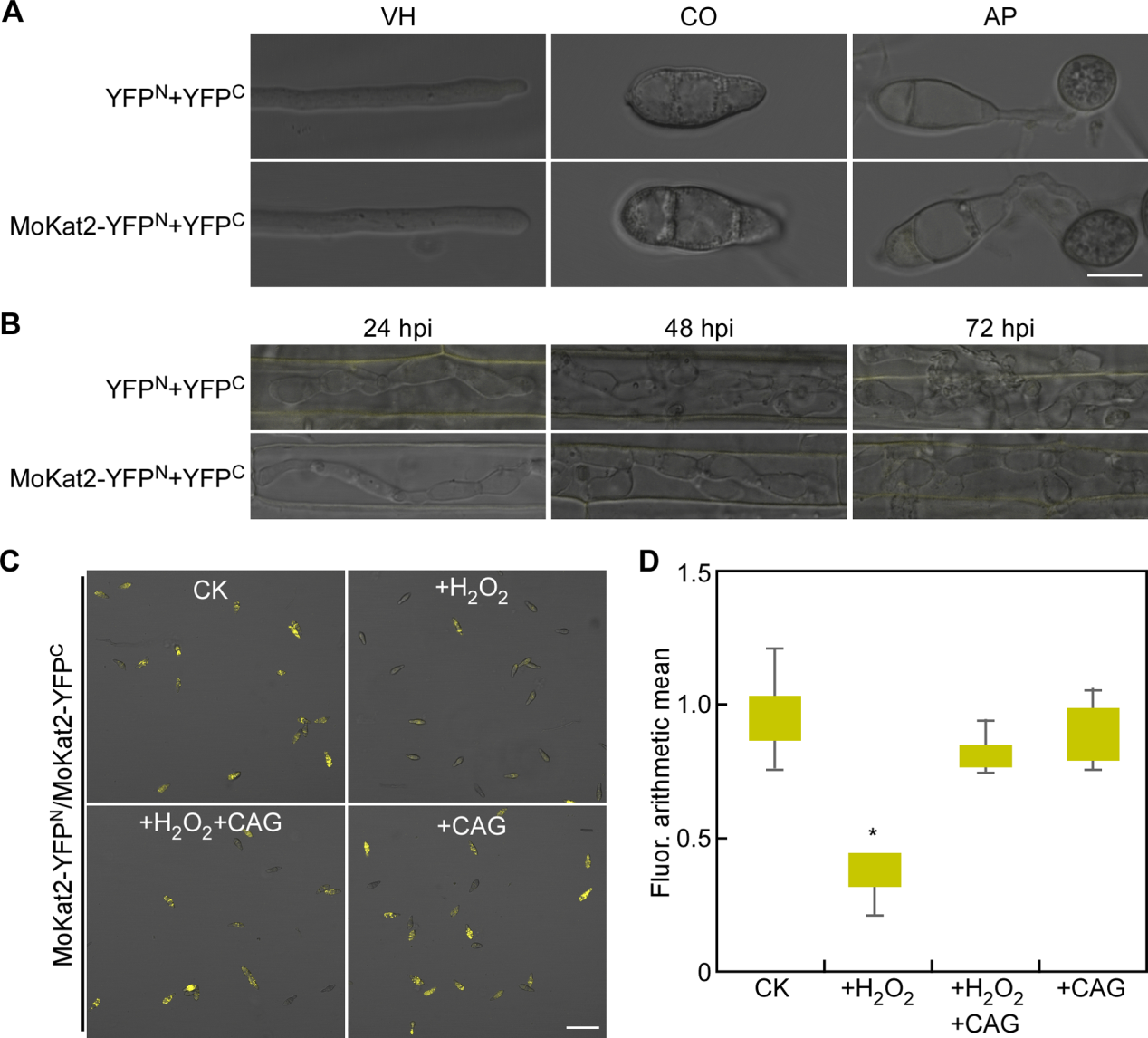


**Figure S7. MoKat2 is dissociated under exogenous H_2_O_2_ treatment. (A and B)** Negative controls for BiFC assay: YFP signals were examined in HY, CO, AP an IH expressing YFP^N^/YFP^C^ empty vectors or MoKat2-YFP^N^/YFP^C^ vectors. Bar=10 μm. (C) YFP signals were examined in conidia treated with or without 5mM H_2_O_2_ and CAG. In addition, conidia were treated with 5mM H_2_O_2_ and then treated by CAG. Bar=50 μm. (D) Statistical analysis of the fluorescence arithmetic mean in conidia. Statistical analysis was performed using GraphPad Prism 8.0.1 and ZEN blue. Asterisk represents significant difference at *p*<0.01.
