# Supplementary material for: Peroxisome dynamics determines host-derived ROS accumulation and infectious growth of the rice blast fungus
Source: mBio. 2023 Nov 15;14(6):e02381-23. doi: 10.1128/mbio.02381-23 (PMC10746245; doi:10.1128/mbio.02381-23)
Supplement: Table S1 — Summary of the sequencing and assembly results, and data filtering of the sequencing results. [file mbio.02381-23-s0008.docx]

Table S1-1. Summary of the sequencing and assembly results.

| Sample | Raw read No. | Raw bases | Raw Q30 No. | Raw N rate | Raw Q20 rate | Raw Q30 rate |
| --- | --- | --- | --- | --- | --- | --- |
| CK1 | 47852632 | 7177894800 | 6813576968 | 0.002137 | 98.27 | 94.92 |
| CK2 | 44643328 | 6696499200 | 6365986926 | 0.002207 | 98.3 | 95.06 |
| +H_2_O_2__1 | 44470206 | 6670530900 | 6350778485 | 0.00209 | 98.39 | 95.2 |
| +H_2_O_2__2 | 50815756 | 7622363400 | 7256346925 | 0.002171 | 98.36 | 95.19 |
| IH24h_1 | 47284280 | 7092642000 | 6654149217 | 3.7e-05 | 97.43 | 93.81 |
| IH24h_2 | 50372708 | 7555906200 | 7083089503 | 3.5e-05 | 97.48 | 93.74 |

Reads No: total number of reads；Bases (bp): total number of bases；Q30 (bp): the total number of bases with the base recognition accuracy rate of more than 99.9%; N(%): the percentage of vague bases; Q20(%): the percentage of bases with the base recognition accuracy rate of more than 99%; Q30(%): the percentage of bases with base recognition accuracy of 99.9% or more.

Table S1-2. Data filtering of the sequencing results.

| Sample | Clean reads No. | Clean data (bp) | Clean reads (%) | Clean data (%) |
| --- | --- | --- | --- | --- |
| CK1 | 45518520 | 6827778000 | 95.12 | 95.12 |
| CK2 | 42505112 | 6375766800 | 95.21 | 95.21 |
| +H_2_O_2__1 | 42458070 | 6368710500 | 95.47 | 95.47 |
| +H_2_O_2__2 | 48278922 | 7241838300 | 95 | 95 |
| IH24h_1 | 25535112 | 3830266800 | 54 | 54 |
| IH24h_2 | 28137242 | 4220586300 | 55.85 | 55.85 |

Clean reads No: the number of clean reads; Clean data (bp): Bases No. of clean reads; Clean reads %: the percentage of clean reads in sequencing reads; Clean data %: the percentage of clean data in sequencing bases.
