# Supplementary material for: Peroxisome dynamics determines host-derived ROS accumulation and infectious growth of the rice blast fungus
Source: mBio. 2023 Nov 15;14(6):e02381-23. doi: 10.1128/mbio.02381-23 (PMC10746245; doi:10.1128/mbio.02381-23)
Supplement: Table S2 — Prediction of the protein subcellular localization and phenotypic analysis of Guy11, ∆Mokat2, MoKAT2-C, and MoKAT2M strains. [file mbio.02381-23-s0009.docx]

Table S2-1. Prediction of the protein subcellular localization.

| Gene | ID | Site | Distance | Identity | Comments |  |  |  |  |
| --- | --- | --- | --- | --- | --- | --- | --- | --- | --- |
| MGG_09512 | THIK_CANTR | Pero | 347.067 | 50% | Peroxisomal | | |  |  |
| MGG_13647 | OTC_TRAHI | Mito | 372.997 | 17.18% | Mitochondrial | | |  |  |
| MGG_10700 | DHSB_USTMA | Mito | 472.058 | 14.92% | Mitochondrial inner membrane | | |  |  |
| MGG_06561 | ILV5_NEUCR | Mito | 343.051 | 16.35% | Mitochondrial | | |  |  |
| MGG_04956 | GLYC_YEAST | Cyto | 275.196 | 16.29% | Cytoplasmic | | |  |  |
| MGG_17054 | PSY_NEUCR | Plas | 530.981 | 16.55% | Integral membrane protein | | |  |  |
| MGG_06148 | EF1A_ABSGL | Cyto | 364.938 | 14.29% | Cytoplasmic | | |  |  |
| MGG_06332 | MPCP_YEAST | Mito | 424.734 | 18.68% | Integral membrane protein.  Mitochondrial inner membrane.  Mitochondrial membrane | | |  |  |

Note: The prediction website: https://wolfpsort.hgc.jp/

Table S2-2. Phenotypic analysis of Guy11, ∆*Mokat2*, *MoKAT2-C* and *MoKAT2^M^* strains

| Strain | Colony diameter (cm)^a^ | | Conidiation (🞨10^4^/cm^2^)^b^ | Appressorium formation (%)^c^ | Collapsed appressorium (%)^d^ | | | |
| --- | --- | --- | --- | --- | --- | --- | --- | --- |
|  | CM | MM |  |  | 1 M | 2 M | 3 M | 4 M |
| Guy11 | 5.1±0.1 | 4.4±0.1 | 13.0±0.4 | 95.9±1.3 | 12.0±5.3 | 30.6±4.6 | 65.3±1.5 | 88.7±4.2 |
| ∆*Mokat2* | 4.9±0.2 | 1.8±0.1* | 9.8±0.8* | 94.7±1.7 | 49.3±4.6* | 66.7±1.2* | 79.3±4.6* | 88.6±3.1 |
| *MoKAT2-C* | 5.2±0.1 | 4.3±0.1 | 12.5±1.1 | 95.4±1.2 | 20.0±4.0 | 33.3±2.3 | 66.7±4.2 | 89.3±3.0 |
| *MoKAT2^M^* | NA | 2.2±0.2* | NA | NA | 54.7±6.4* | 68.6±5.7* | 86.0±2.0* | 89.7±3.1 |

±standard deviation (±SD) was calculated from three repeated experiments, and asterisks indicate significant differences at *P*<0.01.

a. Colony diameter of the indicated strains on CM and MM media after 7 days incubation at 28°C.

b. Quantification of the conidial production of the indicated strains from 10 days SDC cultures.

c. Appressorium formation on hydrophobic surfaces at 24 h post-inoculation (hpi).

d. Percentage of collapsed appressorium under the treatment of 1-4 M glycerol.
