# Supplementary material for: Peroxisome dynamics determines host-derived ROS accumulation and infectious growth of the rice blast fungus
Source: mBio. 2023 Nov 15;14(6):e02381-23. doi: 10.1128/mbio.02381-23 (PMC10746245; doi:10.1128/mbio.02381-23)
Supplement: Table S3 — Primers used in this study. [file mbio.02381-23-s0010.docx]

Table S3. Primers used in this study.

| Primer name | Sequence （5’-3’） | Remark |
| --- | --- | --- |
| KAT2-F1 | GGGGTACC GAAGGCGATACGGCAGACACAC | Amplification of *MoKAT2* 5’ flank sequence |
| KAT2-R2 | CCGGAATTC GTTGTCTTGCGCGGGTTCTTCGG | Amplification of *MoKAT2* 5’ flank sequence |
| KAT2-F3 | GGACTAGT AGTAGAGTAATCAAGCCATGTTGAT | Amplification of *MoKAT2* 3’ flank sequence |
| KAT2-R4 | CGAGCTC TTTTTGCTCATCTCATTGGCATGTC | Amplification of *MoKAT2* 3’ flank sequence |
| KAT2-koin-F | ACCCAATGCTAACCTCCCG | Validation of *MoKAT2*deletion transformants |
| KAT2-koin-R | CGCCGATACCGATCTCAATC | validation of *MoKAT2* deletion transformants |
| KAT2koout-F | CGGCTACGAGTGGAAGGGTT | Validation of *MoKAT2* deletion transformants |
| HphYZRR | CGCTACTGCTACAAGTGGGGCT | Validation of *MoKAT2* deletion transformants |
| KAT2C-F | ACTCACTATAGGGCGAATTGGGTACTCAAATTGGTTCAGGTTCACAGACCCGAAG | *MoKAT2* complementation（native promoter） |
| KAT2C-R | CACCACCCCGGTGAACAGCTCCTCGCCCTTGCTCACCTCTGCAACCCAGAGCG | *MoKAT2* complementation（native promoter） |
| RP27-KAT2C-F | TTTCGTAGGAACCCAATCTTCAAAATGGGTGGTAAGCGATCTATTC | *MoKAT2* complementation（*RP27* promoter） |
| RP27-KAT2C-R | CACCACCCCGGTGAACAGCTCCTCGCCCTTGCTCACCTCTGCAACCCAGAGCG | *MoKAT2* complementation（*RP27* promoter） |
| KAT2-Probe-F | GCTCATTGTTCGGACTGC | Amplification of *MoKAT2* probe sequence |
| KAT2-Probe-R | TCGTAAACGCTGCTCATAT | Amplification of *MoKAT2* probe sequence |
| BDKAT2F | TCAGAGGAGGACCTGCATATGATGGGTGGTAAGCGATCTATTC | Construction of  pGBKT7-*MoKAT2* vector |
| BDKAT2R | TCGACGGATCCCCGGGAATTCTTACTCTGCAACCCAGAGC | Construction of  pGBKT7-*MoKAT2* vector |
| HisKAT2F | GCCATGGCTGATATCGGATCCATGGGTGCCCTTGACAGA | Construction of  pET32a-*MoKAT2* vector |
| HisKAT2R | TTGTCGACGGAGCTCGAATTCTTACTCTGCAACCCAGAGC | Construction of  pET32a-*MoKAT2* vector |
| GSTKAT2F | GATCTGGTTCCGCGTGGATCCATGGGTGCCCTTGACAGA | Construction of  pGEX-4T-2-*MoKAT2* vector |
| GSTKAT2R | CTCGAGTCGACCCGGGAATTCCTTACTCTGCAACCCAGAGC | Construction of  pGEX-4T-2-*MoKAT2* vector |
| FL4362ActinF | CCATGTACCCTGGTCTTTCG | qRT-PCR analysis of *ACTIN* gene |
| FL4362ActinR | TTCGAGATCCACATCTGCTG | qRT-PCR analysis of *ACTIN* gene |
| KAT2FlagF | CTATAGGGCGAATTGGGTACTCAAATTGGTTCAGGTTCACAGACCCGAAG | Construction of pHZ126-*MoKAT2* vector (*with 3×Flag tag*) |
| KAT2FlagR | CTTTATAATCACCGTCATGGTCTTTGTAGTCCTCTGCAACCCAGAGCG | Construction of pHZ126-*MoKAT2* vector (*with 3×Flag tag*) |
| KAT2Ahs-F2 | GAACCCGCGCAAGACAACATGGGTGGTAAGCGATCTATTC | Construction of pYF11*-MoKAT2*^∆^*^AHs^* vector (*with GFP tag*) |
| KAT2Ahs-R1 | GTTGTCTTGCGCGGG | Construction of pYF11*-MoKAT2*^∆^*^AHs^* vector (*with GFP tag*) |
| qRTKAT2F | CCCTTGACAGACTCCAGCAGATTG | qRT-PCR analysis of *MoKAT2* gene |
| qRTKAT2R | GGCAGGCGGTGACAACGATATC | qRT-PCR analysis of *MoKAT2* gene |
| phz65KAT2F | CGACTCACTATAGGGCGAATTGGGTACTCAAATTGCAGGTTCACAGACCCGAAG | Construction of pHZ65-*MoKAT2* vector |
| phz65KAT2R | GCTCACCATCGTGGCGATGGAGCGCTCTGCAACCCAGAGCG | Construction of pHZ65-*MoKAT2* vector |
| phz68KAT2F | CGACTCACTATAGGGCGAATTGGGTACTCAAATTGCAGGTTCACAGACCCGAAG | Construction of pHZ68-*MoKAT2* vector |
| phz68KAT2R | GTTCGGGATCTTGCAGGCCGGGCGCTCTGCAACCCAGAGCG | Construction of pHZ68-*MoKAT2* vector |
| Pyf11KAT2RFPF | ACTCACTATAGGGCGAATTGGGTACTCAAATTGGTTGGGCTCGTTATCGGAGC | Construction of pYF11*-MoKAT2* vector (*with RFP tag*) |
| Pyf11KAT2RFPR | GTCCTCGGTGTTGTCCATCTCTGCAACCCAGAGCG | Construction of pYF11-*MoKAT2* vector (*with RFP tag*) |
| KAT2RFPF | CGCTCTGGGTTGCAGAGATGGACAACACCGAGGAC | Construction of pYF11-*MoKAT2* vector (*with RFP tag*) |
| KAT2RFPR | CACCACCCCGGTGAACAGCTCCTCGCCCTTGCTCACTTACTACTGGGAGCCGGAG | Construction of pYF11-*MoKAT2* vector (*with RFP tag*) |
| 3AKAT2FI | ATGGGTGCCCTTGACAGACTC | Point-mutation of *MoKAT2* |
| 3AKAT2RI | GCATCTCGGCGGCGCCA | Point-mutation of *MoKAT2* |
| 3AKAT2F2 | TGGCGCCGCCGAGATGC | Point-mutation of *MoKAT2* |
| 3AKAT2R2 | TTACTCTGCAACCCAGAGCGC | Point-mutation of *MoKAT2* |
| 3AKAT2F3 | AGACGAGCACGCACGGTT | Point-mutation of *MoKAT2* |
| 3AKAT2R3 | AACCGTGCGTGCTCGTCT | Point-mutation of *MoKAT2* |
| 3AKAT2F4 | AAAGAACACCCGCGGGCGCCGCGGCGTTTGCAGCTTC | Point-mutation of *MoKAT2* |
| 3AKAT2R4 | GAAGCTGCAAACGCCGCGGCGCCCGCGGGTGTTCTTT | Point-mutation of *MoKAT2* |
| *MoKAT2^M^-*F1 | ACTCACTATAGGGCGAATTGGGTACTCAAATTGGTTCAGGTTCACAGACCCGAAG | pYF11-*MoKAT2^M^* vector (*with GFP tag*) |
| *MoKAT2^M^-*R1 | ACCAGACGAGGCCTGACGGTTG | pYF11-*MoKAT2^M^* vector (*with GFP tag*) |
| *MoKAT2^M^-*F2 | CAACCGTCAGGCCTCGTCTGGT | pYF11-*MoKAT2^M^* vector (*with GFP tag*) |
| *MoKAT2^M^-*R2 | CACCACCCCGGTGAACAGCTCCTCGCCCTTGCTCACCTCTGCAACCCAGAGCG | pYF11-*MoKAT2^M^* vector (*with GFP tag*) |
| qRT06332F | CGGAAAGAGTAACCTGAAGCCTTGG | qRT-PCR analysis of MGG_06332 |
| qRT06332R | GACCGACCTTGGCAACGATGAG | qRT-PCR analysis of MGG_06332 |
| qRT06148F | CCAAGGAGGGCGTCAAGTACAAC | qRT-PCR analysis of MGG_06148 |
| qRT06148R | GCAAGTCAGGAGGCATCACAGTC | qRT-PCR analysis of MGG_06148 |
| qRT06561F | TGCGAGTCTTAGGCTCTCCAGTG | qRT-PCR analysis of MGG_06561 |
| qRT06561R | ATGGCAACCTCCTCCTCTGTGAG | qRT-PCR analysis of MGG_06561 |
| qRT13467F | ACGGATTCCATCATGGGCAAGTTC | qRT-PCR analysis of MGG_13467 |
| qRT13467R | CACCTCTGCACGTCCTTGTTCTC | qRT-PCR analysis of MGG_13467 |
| qRT10700F | CGACGCCGAGACCAAGATGAAC | qRT-PCR analysis of MGG_10700 |
| qRT10700R | GCACTCGCTCAGACCAGTAACG | qRT-PCR analysis of MGG_10700 |
| qRT17054F | CGGGTCTGTTGAGCAACTACTTCC | qRT-PCR analysis of MGG_17054 |
| qRT17054R | AACCTCCTCAGCGTTGGCAATG | qRT-PCR analysis of MGG_17054 |
| qRT04956F | TGTTGAGCATTCCCTAAAGCAGACC | qRT-PCR analysis of MGG_04956 |
| qRT04956R | AACGATGTATTTGACCGACGAGACC | qRT-PCR analysis of MGG_04956 |
